# Immunotherapy Efficacy in the Initial Lines of Treatment in Advanced Upper Gastrointestinal Malignancies: A Systematic Review of the Literature

**DOI:** 10.1093/jncics/pkab088

**Published:** 2021-11-16

**Authors:** Konstantinos Kamposioras, Panagiotis Ntellas, Michail Nikolaou, Theodora Germetaki, Ioanna Gazouli, Katerina Dadouli, George Zarkavelis, Anna-Lea Amylidi, Maria Tolia, Davide Mauri

**Affiliations:** 1 Department of Medical Oncology, The Christie NHS Foundation Trust, Manchester, UK; 2 Department of Medical Oncology, University Hospital of Ioannina, Ioannina, Greece; 3 Department of Medical Oncology, Agios Savvas Anticancer Hospital, Athens, Greece; 4 Laboratory of Hygiene and Epidemiology, Faculty of Medicine, University of Thessaly, Larissa, Greece; 5 Department of Radiotherapy, School of Medicine, University of Crete, Heraklion, Greece

## Abstract

**Background:**

The therapeutic role of immune checkpoint inhibitors (ICIs) has represented the cutting edge of clinical research in upper gastrointestinal (GI) malignancies, with these agents now included in the armamentarium of treatment options for advanced gastric and esophageal cancers.

**Methods:**

We performed a systematic literature review and pooled analysis to map out the currently available robust clinical evidence for the use of ICIs in upper GI cancers. Immunotherapy (IO), either as monotherapy or in combination with chemotherapy, and its role in first-line, maintenance, and second-line settings, as well as in specific clinical and biological subgroups, were critically appraised. All statistical tests were 2-sided.

**Results:**

ICIs, in combination with chemotherapy, have provided statistically significant overall survival benefit in the first-line setting in gastric and gastro-esophageal adenocarcinomas (hazard ratio [HR] = 0.83, 95% confidence interval [CI] = 0.76 to 0.90, *P* < .001; based on 4 studies) and esophageal squamous cell carcinoma (HR = 0.72, 95% CI *=* 0.64 to 0.81, *P* < .001; based on 3 studies), albeit with heterogeneous efficacy according to biomarker expression. Patients with esophageal squamous cell carcinoma, and in particular high programmed cell death ligand-1 expression, derive survival benefit when treated with IO in the second-line setting (HR = 0.74, 95% CI = 0.68 to 0.82, *P* < .001; for any level of programmed cell death ligand-1 expression). Clinical trials interrogating the combination of IO with chemotherapy in second-line treatment should be seriously considered in upper GI adenocarcinomas. The role of maintenance IO after initial disease control is still unclear and cannot be recommended. Impressive response rates and survival benefit from IO have been reported in patients with microsatellite instability-high tumors (HR = 0.33, 95% CI = 0.19 to 0.57, *P* < .001), and this warrants further prospective biomarker-driven studies.

**Conclusions:**

IO is changing the treatment landscape in upper GI malignancies. The rapidly developing evidence in the field needs to be critically appraised while further validation of the existing information from ongoing trials is awaited.

Upper gastrointestinal (GI) tract malignancies include cancers of the esophagus, gastro-esophageal junction (GEJ), and stomach. Gastric and GEJ cancers are the fifth and esophageal cancer the sixth leading causes of cancer-related deaths globally (1). Chemotherapy remains the mainstay treatment option for the management of advanced disease, but clinical benefit and survival outcomes remain disappointing. Platinum compounds in combination with a fluoropyrimidine remain the backbone of chemotherapy in the first-line setting in conjunction with trastuzumab in Her-2 expressing tumors (2). In the majority of case s, the disease will progress within a median of 6 to 12 months, and thereafter, depending on the patient’s fitness, second- or even third-line treatment with irinotecan, taxanes, ramucirumab, or trifluridine/tipiracil can be considered (3-7).

Improving the survival outcomes of patients with upper GI malignancies therefore remains an unmet need. Recently, immunotherapy (IO), in particular immune checkpoint inhibitors (ICIs), has been shown to confer statistically significant survival advantage for the treatment of a variety of tumor types. The use of ICIs in upper GI malignancies is a rapidly evolving and cutting-edge field of clinical research. Evidence from randomized controlled clinical trials of their clinical application has grown notably over the last few years and is expected to develop rapidly in the near future. For instance, treatment with ICI anti-PD1, nivolumab, has proven its superiority over placebo in third-line treatment in gastric and GEJ cancers, reducing the risk of death by 37% (8). Although the role of IO in earlier lines of treatment remains unclear, there have been promising preliminary results from early-phase trials (9). In this systematic review, we aim to present a comprehensive summary of evidence from randomized clinical trials of the current state of the art for the use of ICIs in upper GI cancers in the initial lines of treatment and their potential future projections.

## Methods

### Search Strategy and Selection Criteria

We performed an extensive and systematic literature search including both published (full articles) and unpublished (conferences communication, trials declared, medical libraries) material to identify randomized clinical trials reporting data on the use of IO in the first-line, second-line, and maintenance treatment for advanced or metastatic upper GI malignancies. Clinical trials on third-line treatment and beyond were excluded. For further details of our search methodology, please see the step-wise approach description and CONSORT diagram summarized in Supplementary Methods and Supplementary Figure 1 (available online), respectively. All relevant data from the included randomized clinical trials were extracted, and in the case where the same information was available across different trials, pooled analysis was performed to provide the maximum available level of comprehensive evidence and corresponding caveats.

### Pooled Data Statistical Analysis

Summary data methodology meta-analysis of available trials was used for the analyses. The Inverse-Variance and the Mantel-Haenszel statistical methods were applied for calculation of pooled hazard ratios (HRs) and odds ratios (ORs), respectively. Between-studies heterogeneity was evaluated with Cochran’s Q test; in case of statistically significant heterogeneity (Q test *P* < .1), the random effects model was reported; otherwise the fixed effects model was adopted to estimate the pooled ratios. *I*^2^ statistic was also calculated to assess overall heterogeneity.

All 95% confidence intervals (CIs) were used for the analysis. For studies that reported CIs other than 95%, the 95% CIs were calculated and used in the pooled analysis. Statistical significance was set at the 2-sided .05 level. RevMan software V5.4 was used for the completion of the pooled data analysis.

## Results

### Critical Appraisal of the Current Evidence

In total, we identified 6 first-line (10-15), 8 second-line (16-23), and 3 studies investigating the use of IO with ICIs as maintenance treatment (24-26) for upper GI cancers. The majority of these were phase III clinical trials, with obvious variations in the patient populations enrolled and heterogeneity in the programmed cell death ligand-1 (PD-L1) expression levels assessed (Table  1).

**Table 1.** Details of randomized studies in early lines of treatment in advanced upper GI malignancies

Study	Non-Asian/Asian, %	Line of treatment	Phase	Tumor site histology	Arms	No. of patients	PD-L1 expression	Primary endpoints	Secondary endpoints
KEYNOTE 062 NCT02494583	76/24	1st	III	G/GEJ AdenoCa	Pembrolizumab Pembro/Cis/5-Fu Pl+ Cis/5-Fu	256 257 250	CPS ≥ 1	OS (CPS ≥ 1, 10)^a^ PFS (CPS ≥ 1)	ORR, DOR, safety, tolerability, HRQL
ATTRACION-4 NCT02746796	0/100	1st	III	G/GEJ AdenoCa	Nivo/Chemo Pl + SOX/CapOx	362 362	Any	OS, PFS^a^	PFS^b^, ORR, DOR, DCR, TTR, BOR, safety
CheckMate-649 NCT02872116	75/25	1st	III	G/GEJ/E AdenoCa	Nivo/Chemo Folfox/CapOx	789 792	Any	OS, PFS^a^ (CPS ≥ 5)	OS (CPS ≥ 10, 1, or all), PFS (CPS ≥ 10, 1, or all) ORR
KEYNOTE-590 NCT03189719	47.5/52.5	1st	III	E/GEJSiewert I AdenoCa/SCC	Pembro/Chemo Pl+ Cis/5-Fu	373 376	Any	OS, PFS^b^	ORR
CheckMate-648 NCT03143153	30/70	1st	III	Esophageal SCC	Nivo/Chemo Nivo/Ipi Cis/5-Fu	321 325 324	Any	OS, PFS^c^ (CPS ≥ 1)	OS, PFS (all randomized) ORR (CPS ≥ 1 and all)
ESCORT-1st NCT03691090	—	1st	III	Esophageal SCC	Camrelizumab/Chemo Pac/Cis	298 298	Any	OS, PFS^a^	PFS^b^, ORR, DCR, DoR, OS rate, safety, HRQoL
JAVELIN-100 NCT02625610	77/23	Maintenance	III	G/GEJ AdenoCa	Avelumab Folfox/Capox	249 250	Any	OS OS PD-L1 +	PFS
Bang et al. NCT01585987	46.5/53.5	Maintenance	II	G/GEJ AdenoCa	Ipi BSC^c^	57 57	NA	irPFS^a^	PFS, OS, irBORR
PLATFORM NCT02678182	NA	Maintenance	II	E/G/GEJ AdenoCa	Durvalumab Surveillance	105 100	Any	PFS	TTF, ORR, OS, toxicity
ESCORT NCT03099382	0/100	2nd	III	Esophageal SCC	Camrelizumab Doc/Iri	228 220	Any	OS	PFS, ORR, HRQL
ATTRACTION-3 NCT02569242	4/96	2nd	III	Esophageal SCC	Nivo Pac/Doc	210 209	Any	OS	OR, irBORR, PFS, DCR, TTR, DOR
KEYNOTE-181 NCT02564263	61.5/38/5	2nd	III	Esophageal AdenoCa/SCC	Pembrolizumab Pac/Doc/Iri	314 314	Any	OS (CPS ≥ 10, SCC, all)	PFS, ORR (CPS ≥ 10, SCC, all) safety tolerability
KEYNOTE-061 NCT02370498	73/27	2nd	III	G/GEJ AdenoCa	Pembrolizumab Pac	196 199	CPS ≥ 1	OS, PFS^a^ (CPS ≥ 1)	ORR, DOR (CPS ≥ 1, all)^a^^,^^b^, OS, PFS (all)^a^^,^^b^, TTP, safety
KEYNOTE-063 NCT02370498	0/100	2nd	III	G/GEJ AdenoCa	Pembrolizumab Pac	47 47	CPS ≥ 1	OS, PFS	ORR, safety
RATIONALE-302 NCT03430843	21/79	2nd	III	Esophageal SCC	Tislelizumab Pac/Doc/Iri	256 256	Any	OS in All	OS (CPS ≥ 10), PFS, ORR, DoR, safety
Kelly et al. 2019 NCT02340975	55/45	2nd and 3rd	Ib/II	G/GEJ AdenoCa	2L D + T 2L D 2L T 3L D + T 2L/3L D+TIFNγ+	27 24 12 25 19	Any	ORR, PFS at 6 mo	Safety, DCR, DOR, OS PFS
CheckMate-032 NCT01928394	95/NA	2nd and beyond (1 patient in 2nd line)	I/II	Esophageal or G/GEJ AdenoCa	NIVO3 NIVO1 + IPI3 NIVO3 + IPI1	59 49 52	Any	ORR^a^	OS, PFS, DOR, safety

a Central assessment. AdenoCa = adenocarcinoma; BOR = best overall response; BSC = Best Supportive Care; CapOx = capecitabine/oxaliplatin; Chemo = chemotherapy; Cis = cisplatin; CPS = combined positive score; D = durvalumab; DCR = disease control rate; Doc = docetaxel; DOR = duration of response; E = Esophageal; 5-Fu = 5-fluorouracil; G = gastric; GEJ = gastro-esophageal junction; HRQL = health-related quality of life; irBORR = immune-related best overall response rate; Ipi = ipilimumab; Iri = irinotecan; irPFS = immune-related progression-free survival; irTTP = immune-related time to progression; 2L = second line; 3L= third line; NA = not applicable; Nivo = nivolumab; ORR = overall response rate; OS = overall survival; Pac = paclitaxel; PD-L1 = programmed cell death ligand-1; PFS = progression-free survival; Pl = placebo; SCC = squamous cell carcinoma; SOX = S1 (tegafur-gimeracil-oteracil potassium)/oxaliplatin; T = tremelimumab; TTF = time to treatment failure; TTP = time to progression; TTR = time to relapse.

b Investigator’s assessment.

c Total of 79% received chemotherapy with capecitabine, 5-fluorouracil, or S-1.

### First-Line Treatment

Six studies compared ICIs (anti-PD1: nivolumab, pembrolizumab, or camrelizumab) in combination with chemotherapy vs chemotherapy alone (Table  2; Supplementary Tables 1 and 2, available online) (10-15). Three of these studies have been presented at the European Society of Medical Oncology Virtual Congress 2020 (September 19-21, 2020) (11-13) and 2 in the 2021 American Society of Clinical Oncology Annual Meeting (June 4-8, 2021) (14,15) with full peer-reviewed publications still awaited. In the KEYNOTE-062 trial, single-agent pembrolizumab was compared with chemotherapy (10). In most of the studies, only patients with G or GEJ adenocarcinoma were enrolled; however, patients with esophageal adenocarcinoma were also included in CheckMate-649. KEYNOTE-590, CheckMate-648, and ESCORT-1st trials included patients with esophageal squamous cell cancer (ESCC) (11,14,15).

**Table 2.** Efficacy outcomes for first-line use of IO vs chemotherapy for the treatment of upper GI carcinomas

Setting^a^	No. of studies^b^	Study	Investigational agent	OS		PFS		Response rate	
				HR (95% CI)	*P* ^c^	HR (95% CI)	*P* ^c^	OR (95% CI)^d^	*P* ^c^
IO vs Chemo									
PD-L1 CPS > 1%	1	KEYNOTE-062	Pembrolizumab	0.91(0.74 to 1.10)^d^	−	1.66 (1.37 to 2.01)	−	0.29 (0.19 to 0.45)	<.001
PD-L1 CPS > 5% or 10%	1	KEYNOTE-062	Pembrolizumab	0.69(0.49 to 0.97)	−	1.10 (0.79 to 1.51)	−	0.55 (0.29 to 1.04)	.065
IO+Chemo vs Chemo									
All studies	6	KEYNOTE-062	Pembrolizumab	0.85 (0.70 to 1.03)	−	0.84 (0.70 to 1.02)	−	1.60 (1.12 to 2.28)	−
		KEYNOTE-590	Pembrolizumab	0.73 (0.62 to 0.86)	−	0.65 (0.55 to 0.76)	−	1.98 (1.47 to 2.68)	−
		ATTRACTION-4	Nivolumab	0.90 (0.75 to 1.07)	−	0.68 (0.54 to 0.86)	−	1.48 (1.10 to 1.98)	−
		CheckMate-649	Nivolumab	0.80 (0.68 to 0.94)^e^	−	0.77 (0.68 to 0.87)	−	1.84 (1.38 to 2.45)	−
		CheckMate-648	Nivolumab	0.74 (0.59 to 0.94)	−	0.81 (0.67 to 0.99)	−	—	−
		ESCORT-1st	Camrelizumab	0.70 (0.56 to 0.87)	−	0.56 (0.46 to 0.68)	−	—	−
		Pooled evidence	Any agent	0.79 (0.74 to 0.85)	<.001	0.72 (0.64 to 0.81)	<.001	1.72 (1.48 to 2.00)^(4)^	<.001
PD-L1 CPS > 1%	4	Pooled evidence	Any agent	0.54 (0.37 to 0.78)	<.001	0.69 (0.58 to 0.83)	<.001	1.60 (1.12 to 2.28)^(1)^	.009
PD-L1 CPS > 5 or 10%	4	Pooled evidence	Any agent	0.69 (0.61 to 0.77)	<.001	0.62 (0.55 to 0.69)	<.001	1.83 (1.42 to 2.37)^(2)^	<.001
Any PD-L1 status	5	Pooled evidence	Any agent	0.78 (0.73 to 0.84)	<.001	0.69 (0.55 to 0.78)	<.001	1.70 (1.38 to 2.10) ^(2)^	<.001
Squamous histology	3	Pooled evidence	Any agent	0.72 (0.64 to 0.81)	<.001	0.69 (0.65 to 0.79)	<.001	NA	−
Adenocarcinoma histology	4	Pooled evidence	Any agent	0.83 (0.76 to 0.90)	<.001	0.76 (0.70 to 0.83)	<.001	NA	−
MSI-high population (PD-L1–positive population)									
AnyIO vs Chemo	2	Pooled evidence	Any agent	0.33 (0.19 to 0.57)	<.001	(PD-L1 CPS > 1% in KEYNOTE-062 and PD-L1 CPS > 5% in CheckMate-649)			
IO+Chemo vs Chemo	2	Pooled evidence	Any agent	0.35 (0.18 to 0.59)	.002	−	NA	NA	−
IO vs Chemo	1	KEYNOTE-062	Pembrolizumab	0.29 (0.11 to 0.81)	<.001	−	NA	NA	−

a Detailed description of the studies included in the pooled analysis is included in the Supplementary Methods (available online). Chemo = chemotherapy; CPS = combined positive score; IO = immunotherapy; MSI = MicroSatellite Instability; OS = overall survival; PD-L1 = programmed cell death ligand-1; PFS = progression free survival; SCC = squamous cell carcinoma.

b The number of studies with relative data.

c Inverse-Variance and the Mantel-Haenszel statistical methods were applied for calculation of pooled hazard ratios and odds ratios, respectively. A 2-sided *P* value less than .05 was considered statistically significant.

d Number of studies analyzed in the pooled analysis for response rate.

e Confidence interval for the hazard ratio was 99.2% in the original manuscript and was recalculated as the 95% confidence interval for the present comparisons.

In KEYNOTE-062, patients with G or GEJ adenocarcinoma and PD-L1 expression were randomly assigned between treatment with pembrolizumab or chemotherapy. A high PD-L1 expression, based on a combined positive score (CPS) of 10 or greater, predicted a statistically significant median overall survival (mOS) benefit of almost 7 months for those treated with pembrolizumab compared with chemotherapy (mOS = 17.4 vs 10.8 months, respectively; HR = 0.69, 95% CI = 0.49 to 0.97) (10). However, no overall survival (OS) difference was observed in patients with PD-L1 CPS of 1 or greater, and the use of single-agent IO did not improve progression-free survival (PFS) or response rate. Patients with PD-L1 CPS of 10 or greater had an impressive improvement in the duration of response (DOR) exceeding 19 months (10) (Supplementary Table 2, available online). This remains the only reported study available in this setting, to our knowledge, and therefore, the striking survival benefit and improvement in DOR in patients with high PD-L1 expression will need to be validated in other studies.

On the other hand, the combination of chemotherapy with IO (chemo-IO) has displayed clinically significant improvement in OS both in patients with G or GEJ adenocarcinoma and ESCC. In CheckMate-649, more than 1500 patients with G or GEJ adenocarcinoma were randomly assigned to receive either treatment with nivolumab plus chemotherapy or oxaliplatin, fluoropurimidine. There was improved survival with chemo-IO combination both in the overall population (mOS = 13.8 in the chemo-IO arm vs 11.6 months in the chemotherapy arm; HR = 0.80, 99.3% CI = 0.68 to 0.94) and in the PD-L1–expressing tumors with CPS of at least 1 (mOS = 14 in the chemo-IO arm vs 11.3 months in the chemotherapy arm; HR = 0.77, 99.3% CI = 0.64 to 0.92) (12). Nevertheless, this was not confirmed in the other 3 studies in which chemo-IO was compared with standard chemotherapy (10,11,13). In addition, PFS was statistically improved in 3 of the randomized studies (11-13) but not in the KEYNOTE-062 (10) (Supplementary Table 2, available online).

In ATTRACTION-4, 724 patients received treatment with either chemotherapy alone or nivolumab and chemotherapy. The trial, in which PD-L1 expression was not used for patient selection, did not identify any OS difference between the 2 study arms (HR = 0.90, 95% CI = 0.75 to 1.08) (13), although there was some improvement in the other coprimary endpoint of median PFS (10.45 months in the nivolumab plus chemotherapy arm vs 8.34 months in the chemotherapy arm; HR = 0.68, 98.5% CI = 0.51 to 0.90). It could be argued that the difference in the patient population (only patients from Asia were included), the higher rate of patients treated with second-line treatment, and the lack of biomarker selection may account for the OS discordance between ATTRACTION-4 and CheckMate-649. However, the improvement in PFS will need to be interpreted with caution because these are only results from the interim analysis presented at the European Society for Medical Oncology (ESMO) 2020 congress.

KEYNOTE-590 was the first study to present results on patients with ESCC. The majority (73%) of the enrolled population in this trial (n = 749) had a diagnosis of esophageal squamous cancer. In this particular subgroup, there was a statistically significant OS gain of almost 3 months (mOS = 12.6 vs 9.8 months; HR = 0.73, 95% CI = 0.60 to 0.88, *P *=* *.0006) with the use of the chemo-pembrolizumab combination, which was even more pronounced in the group with high CPS ≥ 10; (mOS = 13.9 vs 8.8 months; HR = 0.57, 95% CI = 0.43 to 0.75, *P *=* *.0001) (11). Patients with ESCC also had better PFS when treated with chemo-IO, and the treatment response rate in this subgroup is expected to be higher when the mature results of the trial become available. Recently, CheckMate-648 and ESCORT-1st confirmed a 3-month survival benefit in patients with ESCC from the chemo-IO combination (14,15) (Table  1; Supplementary Table 1, available online), and actually there was a more than 6-month OS gain when nivolumab was administered with chemotherapy in the PD-L1 ≥ 1 population (mOS = 15.4 vs 9.1 months; HR = 0.54, 95% CI = 0.37 to 0.80, *P *<* *.0001) (15).

CheckMate-648 is the first trial to present results from treatment with the double IO combination (ipilimumab and nivolumab) over chemotherapy alone and showed a 2-month survival improvement in the overall population and 4 months in the biomarker-selected population (CPS ≥ 1) for patients with ESCC (15). Although the absolute benefit from nivolumab plus ipilimumab was not as high as that seen with nivolumab plus chemotherapy, it is worth noticing the crossover of the curves observed in the initial few months of the treatment as well as a longer DOR (11.8 months in nivolumab plus ipilimumab vs 8.4 months in the nivolumab plus chemotherapy arm) in those responding to the double-IO treatment.

Our pooled analysis of the clinical trials results (Table  2; Supplementary Figures 2-4, available online) shows that treatment with chemo-IO in the whole patient population provided survival benefit with a 20% reduction in the risk of death (pooled HR for OS = 0.79, 95% CI = 0.74 to 0.85) compared with chemotherapy alone (Table  2; Supplementary Figure 2, A, available online). Relative reduction in the risk of death was higher (up to 30%) among patients with high PD-L1 expression ie, with CPS greater than or equal to 5 or 10 (HR = 0.69, 95% CI = 0.61 to 0.77) (Table  2; Supplementary Figure 2, C, available online). In addition, survival benefit from the use of combined chemo-IO compared with chemotherapy alone remained statistically significant when cases with ESCC were excluded from analysis (HR = 0.83, 95% CI = 0.76 to 0.90, *P* < .001) and for any PD-L1 CPS subgroups considered (Table  2; Supplementary Figure 2, B and E, available online). Based on the recently presented studies in the American Society of Clinical Oncology (ASCO) 2021 congress, there is robust evidence that chemo-IO reduces the risk of death by 28% when used as first-line treatment in ESCC (HR = 0.72, 95% CI = 0.64 to 0.81, *P* < .001) (Table  2; Supplementary Figure 2, F, available online).

In summary, patients with any histologic type (adenocarcinoma or squamous cell carcinoma) and any PD-L1 CPS expression may benefit and are good candidates for combination chemo-IO in first-line treatment. Similarly, PFS was also higher for chemo-IO irrespective of histology or PD-L1 expression (overall pooled HR = 0.72, 95% CI = 0.64 to 0.81, *P* < .001; for adenocarcinoma only, HR = 0.76, 95% CI = 0.70 to 0.83, *P* < .001; and for ESCC, HR = 0.69, 95% CI = 0.65 to 0.79, *P* < .00001) (Table  2, Supplementary Figure 3, A-F, available online). Chemo-IO improved response rate irrespective of PD-L1 expression (Table  2; Supplementary Figure 4, A-C) compared with chemotherapy alone. This is a notable observation and may allude to the necessity of chemotherapy for potentially prompt tumor shrinkage especially in symptomatic patients, who then could benefit additionally from IO.

Patients with gastric adenocarcinoma seemed to obtain survival benefit similarly to GEJ tumors in the first-line setting (10,12). Nevertheless, the lack of detailed location-specific survival did not allow us to perform pooled analysis based on tumor site. This will need to be further explored in the future pending availability of data. Beyond the location or specific histology, the molecular subtype may also determine the response of upper GI tumors to IO, and it is imperative to gain a much better understanding of the interplay between tumor cells and the tumor (immune-)microenvironment to tailor treatment more effectively (27).

Although we acknowledge that the final results from the 5 first-line studies described above are yet to be published in full, the statistical significance from our pooled analysis is promising, and chemo-IO is expected to have a prominent role in the care for advanced upper GI adenocarcinoma. Indeed, the U.S. Food and Drug Administration has recently approved pembrolizumab in combination with chemotherapy for esophageal and GEJ malignancy regardless of PD-L1 status based on the results from KEYNOTE-590 (28), and the National Comprehensive Cancer Network (NCCN) has altered their recommendations for first-line therapy for gastric cancer to include nivolumab for CPS of 5 or greater (29).

### Maintenance Treatment

The role of maintenance therapy in upper GI cancer remains controversial. We identified 3 clinical trials that randomly assigned patients with Her-2 negative G or GEJ adenocarcinomas with disease control following completion of first-line chemotherapy (Table  3; Supplementary Tables 3 and 4, available online). In the JAVELIN-100 phase III trial, 499 patients with gastric or GEJ adenocarcinoma received maintenance therapy with either IO or chemotherapy (25). In the phase II randomized trial by Bang et al., the control arm was best supportive care, but it is important to note that 80% of the 114 randomly assigned patients ended up receiving chemotherapy in this cohort (24). Thus, we can consider chemotherapy as the control arm for both trials. In both, the use of ipilimumab [a monoclonal anibody against the cytotoxic T-lymphocyte-associated protein-4 (anti-CTLA-4)] (24) or avelumab (anti-PD-L1 ICI) (25) did not demonstrate improvement in the primary study endpoints, namely immune-related PFS (24) and OS (25) compared with chemotherapy alone as maintenance strategy. Similarly, IO did not improve PFS and response rate.

**Table 3.** Efficacy outcomes for the use of IO as maintenance treatment for upper GI carcinomas compared with standard chemotherapy alone^a^

Setting	No. of studies	Study	Investigational agent	Overall survival		PFS		Response rate	
				HR (95% CI)	*P* ^b^	HR (95% CI)	*P* ^b^	OR (95% CI)	*P* ^b^
All studies	3	Bang et al., 2017^c^	Ipilinumab	1.11 (0.68 to 1.80)	−	1.59 (0.90 to 2.81)^d^	−	0.24 (0.03 to 2.19)	−
		JAVELIN-100	Avelumab	0.91 (0.74 to 1.13)	−	1.04 (0.86 to 1.27)	−	0.91 (0.55 to 1.51)	−
		PLATFORM	Durvalumab	0.92 (0.68 to 1.29)	−	0.79 (0.59 to 1.06)	−	−	−
		Pooled evidence	Any agent	0.94 (0.79 to 1.11)	.46	1.02 (0.59 to 1.06)	.92	0.83 (0.51 to 1.36)	.47
Any PD-L1 status	2	Pooled evidence	Any agent	0.94 (0.77 to 1.15)	.55	1.09 (0.90 to 1.31)	.37	0.83 (0.51 to 1.36)	.47
PD-L1 CPS > 1%	1	JAVELIN-100	Avelumab	1.13 (0.57 to 2.23)	−	1.04 (0.53 to 2.02)	−	0.91 (0.55 to 1.51)	−

a Only data for adenocarcinoma were available for analysis. CI = confidence interval; CPS = combined positive score; GI = gastrointestinal; HR = hazard ratio; IO = immunotherapy; OR = odds ratio; PD-L1 = programmed cell death ligand-1; PFS = progression-free survival.

b Inverse-Variance and the Mantel-Haenszel statistical methods were applied for calculation of pooled hazard ratios and odds ratios, respectively. A 2-sided *P* value less than .05 was considered statistically significant.

c Confidence interval for the hazard ratio was 92% in the original manuscript, recalculated to the 95% confidence interval for present comparisons.

d Confidence interval for the hazard ratio was 80% in the original manuscript, recalculated as the 95% confidence interval for present comparisons.

The comparison results of surveillance vs durvalumab after platinum-based induction chemotherapy in patients with esophagogastric adenocarcinoma were recently presented from the PLATFORM trial, a prospective, open-label, multicenter, adaptive phase II study. The study did not meet its primary endpoint, and durvalumab did not improve PFS compared with surveillance after first-line induction treatment with platinum-based chemotherapy (26) despite the initial results suggesting improved radiological responses with the use of the anti-PD-L1 ICI (30).

Understandably, the lack of benefit was confirmed in the pooled analysis from the 3 studies (Table  3; Supplementary Figures 5-7, available online). We also did not identify any particular subgroups of patients in whom IO conferred better survival. In JAVELIN-100, there was only a small number of patients with PD-L1 CPS greater than or equal to 10 included, and hence no definitive conclusions can be made even in this subgroup that may have been expected to have higher probability of benefit from IO. Nonetheless, it should be underscored that the use of anti-CTLA-4 was probably not the most favorable investigational agent for the IO arm in view of its low overall efficacy as monotherapy in the treatment of cancer in general. Thus, the overall efficacy of maintenance IO with anti-PD-L1/PD-1 ICIs might have been underestimated in the summative analysis.

Currently available data do not support the use of IO monotherapy as maintenance therapy following disease control with chemotherapy. It should be noted that although chemotherapy is being broadly used as maintenance treatment (31) and as a comparator in the control arms in upper GI studies, the existing evidence cannot support this approach.

### Second-Line Treatment

Eight reported randomized clinical studies have assessed the use of ICIs in the second-line setting (16-23) (Table  1; Supplementary Tables 5 and 6, available online). In 6 of them, ICIs were compared with chemotherapy (16,17,19,20,22,23). The Kelly et al. (18) and CheckMate-032 studies (21) investigated the use of different IO schedules. In the phase I-II study by Kelly et al. (18), the combination of durvalumab (anti-PD1) plus tremelimumab (anti-CTLA-4) improved OS by almost 6 months compared with single agents durvalumab or tremelimumab (Supplementary Table 5, available online). Nonetheless, there was no standard chemotherapy comparator arm in the study, and no definitive recommendations for its clinical use can yet be made. CheckMate-032 compared nivolumab alone or in combination with ipilimumab in 2 different dosing schedules in pretreated patients, the majority of whom had previously received 2 or more lines of treatment (21). To date, only the results of the cumulative analyses have been presented, and therefore it is impossible to make any interpretation of the efficacy of these ICIs for second-line treatment.

Four phase III trials, namely KEYNOTE-181 (17), ATTRACTION-3 (19), ESCORT (20), and RATIONALE-302 (23), randomly assigned patients with ESCC to either IO or standard chemotherapy in the second-line settings. Patients showed statistically significantly better mOS and longer DOR when treated with IO (Supplementary Tables 5 and 6, available online). The use of the anti-PD1 camrelizumab (ESCORT) increased survival by 2 months compared with chemotherapy (mOS = 8.3 vs 6.2 months; HR = 0.71, 95% CI = 0.57 to 0.87) (20), similar to the clinical benefit observed with the use of nivolumab in ATTRACTION-3 (mOS = 10.9 vs 8.4 months; HR = 0.77, 95% CI = 0.62 to 0.96) (19) and tislelizumab (an anti-PD-1 monoclonal antibody) in RATIONALE-302 (mOS = 8.6 vs 6.3 months; HR = 0.70, 95% CI = 0.57 to 0.85) (23). The survival benefit was more prominent in patients with ESCC and high PD-L1 expression (CPS ≥ 10) as reported in the KEYNOTE-181 study (mOS = 10.3 on IO vs 6.7 months on chemotherapy; HR = 0.64; 95% CI = 0.46 to 0.90) (17) and RATIONALE-302 (mOS =10.3 on IO vs 6.8 months on chemotherapy; HR = 0.54, 95% CI = 0.36 to 0.79). In KEYNOTE-181, the survival was more favorable in patients with ESCC and any PD-L1 expression when treated with IO, but this was not strictly statistically significant as per the prespecified superiority boundaries. In the ESCORT and RATIONALE-302 trials there was a higher response rate (20.2% vs 6.4%) (20) and (20.3% vs 9.8%) (23), respectively, for patients with ESCC treated with IO with statistical improvement in PFS (20). This was, however, not validated in the 2 other studies (17,19).

Intriguingly, there has not been any reported survival benefit in patients with esophageal or G or GEJ adenocarcinoma following second-line treatment with IO (16,17,22) as seen in the KEYNOTE-181 (17), KEYNOTE-061 (16), and KEYNOTE-063 (22) studies (Table  1). The recently updated results of the KEYNOTE-061 trial confirmed that patients with high PD-L1 expression (CPS ≥ 10) had a trend for survival benefit when treated with pembrolizumab, but this was not statistically significant (mOS =10.4 vs 8.0 months; HR = 0.69, 95% CI = 0.46 to 1.05) (32). The study did not present the survival data on the overall population, and it mainly focused on the CPS greater than or equal to 1 subgroup (32). After the initial presentation of the KEYNOTE-061 trial (16), enrolment in KEYNOTE-063 closed prematurely, and hence there was no statistical power to identify differences between the studied groups (22).

In our pooled analysis of all the studies, there was statistically significant improvement in OS (HR = 0.81, 95% CI = 0.74 to 0.88, *P* < .001) without improvement in PFS or ORR (Table  4; Supplementary Figures 8-10, available online). The improvement in OS was predominantly driven by the benefit seen in patients with ESCC (HR = 0.74, 95% CI = 0.68 to 0.82, *P* < .001). Patients with ESCC and CPS greater than or equal to 1 had statistically significant survival benefit from IO (HR = 0.64, 95% CI = 0.51 to 079, *P* < .001), similar to those with CPS greater than or equal to 10 (HR = 0.62, 95% CI = 0.54 to 0.81, *P* < .001). This was not actually the effect of the high CPS, because patients with a score less than 5 still gained statistically significant survival benefit (pooled HR = 0.76, 95% CI = 0.64 to 0.90, *P =* .002) (Table  4; Supplementary Figure 8, J, available online). In the ESCC group with CPS less than 1, there was a non-statistically significant improvement in survival (HR = 0.83, 95% CI = 0.68 to 1.01, *P *=* *.07), and further randomized evaluation in this groups will be needed with the appropriate statistical design. Besides the OS benefit, CPS greater than or equal to 10 predicted for statistically significantly higher PFS (HR = 0.47, 95% CI = 0.24 to 0.88) and response rate (HR = 3.74, 95% CI = 1.50 to 9.33, *P* = .004) and CPS greater than or equal to 1 predicted for higher PFS (HR = 0.60, 95% CI = 0.43 to 0.84), as was initially reported in the ESCORT trial (20). In conclusion, there is compelling evidence from our pooled analysis that IO should be offered as a second-line treatment in patients with ESCC and positive PD-L1 expression.

**Table 4.** Efficacy outcomes for the use of IO in second or more line of treatment for upper GI carcinomas compared with standard chemotherapy

Setting	No. of studies	Study	Investigational agent	Overall survival		PFS		Response rate	
				HR (95% CI)	*P* ^a^	HR (95% CI)	*P* ^a^	OR (95% CI)	*P* ^a^
All studies	6	ATTRACTION-3	Nivolumab	0.77 (0.62 to 0.96)	−	1.08 (0.87 to 1.34)	−	0.87 (0.51 to 1.49)	−
		ESCORT	Camrelizumab	0.71 (0.57 to 0.87)	−	0.69 (0.56 to 0.86)	−	3.72 (1.98 to 6.99)	−
		KEYNOTE-061	Pembrolizumab	0.94 (0.79 to 1.12)	−	1.49 (1.25 to 1.57)	−	0.77 (0.48 to 1.24)	−
		KEYNOTE-063	Pembrolizumab	NA	−	NA	−	0.62 (0.20 to 1.90)	−
		KEYNOTE-181	Pembrolizumab	0.89 (0.75 to 1.05)	−	1.11 (0.94 to 1.31)	−	2.09 (1.21 to 3.64)	−
		RATIONALE-302	Tislelizumab	0.70 (0.57 to 0.85)	−	NA	−	NA	−
Any PD-L1 status	5	Pooled evidence	Any agent	0.81 (0.74 to 0.88)	< .001	1.06 (0.79 to 1.42)	.70	1.31 (0.69 to 2.49)	.41
All SCC	4	Pooled evidence	Any agent	0.74 (0.68 to 0.82)	< .001	0.88 (0.69 to 1.14)	.34	1.98 (0.81 to 4.84)	.13
All AdenoCa	2	Pooled evidence	Pembrolizumab	0.99 (0.85 to 1.15)	.89	1.49 (1.25 to 1.77)	−	0.75 (0.48 to 1.15)	.19
PD-L1 CPS > 1%	3	Pooled evidence	Any agent	0.73 (0.63 to 0.84)	< .001	0.88 (0.43 to 1.79)	.72	1.08 (0.66 to 1.77)	.76
SCC	2	Pooled evidence	Cam/Nivo	0.64 (0.51 to 079)	< .001	0.60 (0.43 to 0.84)^b^	NA	NA	−
Adeno	1	KEYNOTE-061	Pembrolizumab	0.81 (0.66 to 1.00)		1.25 (1.02 to 1.54)	−	1.08 (0.66 to 1.77)	.76
PD-L1 CPS > 10%	5	Pooled evidence	Any agent	0.65 (0.55 to 0.78)	< .001	0.71 (0.56 to 0.89)	.003	3.82 (1.91 to 7.66)	<.001
SCC	4	Pooled evidence	Any agent	0.62 (0.54 to 0.81)	< .001	0.47 (0.24 to 0.88)	−	3.74 (1.50 to 9.33)	.004
AdenoCa	2	Pooled evidence	Pembrolizumab	0.76 (0.54 to 1.07)	.11	0.79 (0.51 to 1.21)	−	3.83 (1.42 to 10.32)	.008
PD-L1 CPS < 1%	3	Pooled evidence	Any agent	0.93 (0.79 to 1.10)	.41	1.27 (0.50 to 3.25)	.62	0.15 (0.03 to 0.72)	.02^c^
PD-L1 CPS < 1% SCC	2	Pooled evidence	Cam/Nivo	0.83 (0.68 to 1.01)	.07	0.79 (0.59 to 1.05)^b^	NA	−	−
PD-L1 CPS < 1% AdenoCa	1	KEYNOTE-061	Pembrolizumab	1.20 (0.89 to 1.63)	−	2.05 (1.50 to 2.79)	−	−	−
PD-L1 CPS < 5% SCC	2	Pooled evidence	Cam/Nivo	0.76 (0.64 to 0.90)	.002	0.78 (0.61 to 0.99)^b^	NA	−	−
PD-L1 CPS < 10% SCC	3	Pooled evidence	Any agent	0.78 (0.67 to 0.90)	.006	0.74 (0.59 to 0.94)^b^	NA	−	−
PD-L1 CPS < 10% all	3	Pooled evidence	Any agent	0.83 (0.68 to 1.02)	.08	NA	−	−	−
MSI-high in PD-L1 positive	1	KEYNOTE-061	Pembrolizumab	0.42 (0.13 to 1.31)	−	NA	−	4.30 (0.70 to 27.16)	−

a Inverse-Variance and the Mantel-Haenszel statistical methods were applied for calculation of pooled hazard ratios and odds ratios, respectively. A 2-sided *P* value less than .05 was considered statistically significant. AdenoCa = adenocarcinoma; Cam = camrelizumab; Chemo = chemotherapy; CPS = combined positive score; GI = gastrointestinal; IO = immunotherapy; MSI = MicroSatellite Instability; Nivo = nivolumab; OS = overall survival; OR = odds ratio; PD-L1 = programmed cell death ligand-1; PFS = progression-free survival; SCC = squamous cell carcinoma.

b These results come only from ESCORT trial.

c Result is based on KEYNOTE-061 trial.

Indeed, the FDA has recently approved pembrolizumab for the treatment of ESCC patients progressing in 1 or more lines of treatment with a high expression PD-L1 (CPS ≥ 10) (33), based predominantly on the results from KEYNOTE-181. The pooled analysis from 3 randomized studies confirmed the benefit offered in this group of patients, and the approval of ICIs in this setting should be considered universally as a standard of care for second-line treatment for ESCC. Nivolumab also has been approved for ESCC irrespective of PD-L1 expression, based on the results of the ATTRACTION-3 study (19). The pooled analysis also confirmed the benefit of IO in patients with ESCC and any CPS expression, albeit with no improvement in PFS or response rate (Table  4). Importantly, in ATTRACTION-3, there was an initial attrition of patents during early follow-up, suggesting that they did worse on IO, which also has been reported in other IO trials (34,35). It could be argued that this group could be considered for chemo-IO to avoid early patient death, especially in symptomatic patients or those with high disease burden.

The pooled analysis confirmed that IO was not superior to chemotherapy in patients with esophageal or G or GEJ adenocarcinoma (HR = 0.99, 95% CI = 0.85 to 1.15) (Table  4; Supplementary Figure 8, E, available online). High CPS greater than or equal to 10 predicted for higher response rate in patients with G or GEJ adenocarcinoma (HR = 3.83, 95% CI = 1.42 to 10.32) but with no OS or PFS improvement. PD-L1 expression did not predict for response to IO in patients with upper GI adenocarcinoma in the early-phase trials (18,21). Patients with G or GEJ adenocarcinoma and high PD-L1 expression similarly did not gain survival benefit from IO in the second-line setting (16). However, apparently there was a signal for better survival in the pooled analysis, and more randomized data are needed before making further conclusions about the use of IO in the adenocarcinoma subpopulation with PD-L1 CPS greater than 10% because only 163 patients overall were available for analysis (75 in the investigational arms and 88 in the experimental arms) (Supplementary Figure 8, F, available online).

### Groups of Special Interest

####  

##### Microsatellite Instability-High (MSI-H) Disease

The presence of a deficient mismatch repair mechanism in cancer cells has been linked to development of a high mutational burden, with an improved response to and survival benefit from ICIs. This led to the accelerated approval of pembrolizumab in MSI-H malignancies (36). The prognostic role of MSI-H expression in early-stage gastric cancer has been previously confirmed in the post hoc analysis of the Medical Research Council Adjuvant Gastric Infusional Chemotherapy (MAGIC) trial (37), whereas there was a remarkable response rate of 71% in the 7 patients treated with pembrolizumab after 2 lines of treatment in the KEYNOTE-059 trial (38). The predictive role of MSI, however, has not been thoroughly explored in earlier lines of treatment.

In the first-line setting, MSI-H has consistently predicted excellent response to IO in patients with PD-L1–expressing tumors in both the CheckMate-649 and KEYNOTE-062 studies (10,12). Nonetheless, overall, only 83 MSI-H patients were randomly assigned in these 2 studies (50 in experimental vs 33 in control arms). In KEYNOTE-062, there was a 71% reduction in the risk of death in patients with CPS greater than or equal to 1 (HR = 0.29, 95% CI = 0.11 to 0.81) and 79% in patients with CPS greater than or equal to 10 (HR = 0.21, 95% CI = 0.06–0.83) (10). Similar reduction in the risk of death (67%) was described in CheckMate-649 (12). The pooled analysis of these 2 studies confirmed the statistically significant survival benefit from chemo-IO in this subgroup of patients (HR = 0.33, 95% CI = 0.19 to 0.57, *P* < .001) as well as IO alone in first line of treatment (Table  2; Supplementary Figure 11, available online). For this reason, despite this group of patients representing only a small population fraction (4%-7%), assessment of MSI status at baseline should be recommended to inform the selection of treatment appropriately and timely.

In the JAVELIN-100 maintenance trial, only a small number of patients had MSI-H disease, and although the confidence interval crossed 1, there might be again a signal towards favorable response in this group (25). Both the KEYNOTE-061 and CheckMate-032 trials suggested a clinical benefit from IO in MSI-H tumors but with insufficient numbers to achieve statistical significance (16,39).

A recent meta-analysis by Pietrantonio et al. (40) confirmed the statistically significant survival benefit observed in the MSI-H population with gastric adenocarcinoma when treated with ICIs. The overall risk reduction for death was 66% (HR = 0.34, 95% CI = 0.21 to 0.54) when all the studies in first and second lines were collectively analyzed. These results are very similar to the pooled analysis we performed for patients receiving IO in the first-line setting. In view of the lack of statistical significance from JAVELIN-100 and KEYNOTE-061, the overall benefit observed in the meta-analysis is driven from the first-line treatment. The MSI-H group of patients warrants further evaluation in well-designed clinical trials, especially in the first-line setting to avoid the dropouts observed due to the rapid decline in fitness of upper GI patients after disease progression. This approach has recently proven particularly successful for patients with colorectal cancer and MSI-H disease who were treated with pembrolizumab in the first-line setting (35).

##### Poor Performance Status

All the studies included patients of good Eastern Cooperative Oncology Group Performance Status (ECOG‐PS 0-1). In the first-line setting, fitness did not predict for better response (10-12). Indeed, patients with ECOG‐PS 1 appeared to have managed marginally better (13). In the second-line setting, although patients with ECOG‐PS 0 seemed to benefit more from IO (16), no difference was observed in the overall population and in those with CPS greater than or equal to 10 in the KEYNOTE-181 study (17), whereas in 2 other studies, patients with ECOG‐PS 1 did better on ICIs (19,20). Retrospective data have suggested that patients with poor performance status (ECOG‐PS ≥ 2) might not benefit from nivolumab (41), but the role of IO in this group of patients needs to be further evaluated systematically, and biomarker-driven studies in this subgroup remain an unmet need.

##### Ethnic Origin

In the majority of studies analyzed, patients of Asian origin had better survival outcomes compared with non-Asian population (10,17,19,25), although this was not confirmed in the KEYNOTE-061 study (16). Cumulative evidence has indicated that Asian patients seem to gain more benefit when treated with anti-PD-1/PD-L1 ICI (42). Although the underlying mechanism to explain this difference remains unclear, some have suggested that this may be due to the difference in inter-population mutational profiles that could be related to IO response (43) or difference in clearance rates affecting the bioavailability of ICI in the body (44). Nevertheless, racial differences in pharmacokinetic studies were not confirmed when ATTRACTION-2 and CheckMate-032 were analyzed (45). It should be also taken into account that the better survival shown in the Asian population could be related to better performance status, potentially linked to earlier diagnosis in that population due to the existence of screening programs and to the higher number of postprogression lines of treatment. These differences will need to be considered in study designs for correct interpretation of the results.

## Discussion

Despite the strengths of this review, certain limitations need to be considered. Most of the trials were designed to show superiority of the investigational agent. However, KEYNOTE-062 was a noninferiority study (10) that could potentially affect the results of the pooled analysis. Certain trials were open-labeled that could have affected the participation of patients on the standard chemotherapy arm (10,16,17). Changes in the study design as in the case of KEYNOTE-061, where patients with CPS less than 1 were excluded after 83% of the patients had been recruited, might have also introduced bias in the final results. In the pooled analysis individual patient data can target outcomes separately by subgroups of patients who have various risks of death (46). The analysis is based on data from trials whose results have been published, and we note that publication bias is a potential threat to the validity of the results. We did not obtain updated individual patient data; the use of such data might have further enhanced the accuracy and reduced the uncertainty of the estimates (47,48). There was heterogeneity in the trial population; hence, we performed subgroup analyses based on histological type, MSI status, and PD-L1 expression but not according to ethnic background because data were limited. The group of patients enrolled varied in terms of CPS status.

Patients with upper GI adenocarcinoma did not benefit from IO alone in the second-line setting, but the combination of chemo-IO seemed to be effective as first-line treatment in this histological subtype. Indeed, when we performed pooled analysis for the treatment of upper GI adenocarcinoma in the second-line setting, there was a trend for better PFS and response rate in the chemotherapy arm (Table  4; Supplementary Figure 10, D). Therefore, the role of chemo-IO in the second-line setting for the treatment of G or GEJ adenocarcinomas needs to be further evaluated to assess the OS benefit and the potentially needed rapid cytotoxic responses in symptomatic patients, who are actually in higher risk of disease progression and death. Combination chemo-IO is potentially a more favorable treatment strategy, because among others, this can lead to eradiation of immune suppressive cells, increased penetration of T-cell into the tumor, and induction of tumor cell death, further eliciting systemic and intra-tumoral immune response (49). These will need to be balanced against the effect of cytotoxic drugs on T-cell population and the immunosuppressive role of the corticosteroids. The results of relevant trials are awaited (50).

So far, the reported studies included patients with Her-2–negative tumors. The combination of IO with anti-Her-2 monoclonal antibodies has provided promising results without raising safety issues (39,51), and the results from phase III randomized trials are expected (52). The role of maintenance treatment in ESCC remains unclear, and this would be an interesting field for future research.

In early-stage disease, the recently presented CheckMate-577 study reported that nivolumab as adjuvant treatment after chemoradiotherapy and surgery for esophageal or GEJ cancer doubled disease-free survival compared with placebo (53). This treatment now has been included in the National Comprehensive Cancer Network (NCCN) guidelines (54) and is expected to be a new standard of care in the adjuvant setting. Further results on the role of IO in perioperative setting are awaited through the KEYNOTE-585 and DANTE trials (55,56), and the combination of pembrolizumab and preoperative chemoradiotherapy (57) for the treatment of esophageal cancer is currently being assessed.

This study is the first systematic review, to our knowledge, to interrogate the use of IO in early treatment lines for advanced or metastatic disease in upper GI malignancies to provide an evidence-based state of the art practice. Multiple pooled analyses were provided to better summarize and present the cumulative evidence when variable information for the same outcome measures, across different trials, was available.

In anticipation of the final results, the initial signal for the combination of IO with chemotherapy seems very promising in the first-line treatment for advanced upper GI adenocarcinomas and ESCC, especially with high PD-L1 expression (CPS ≥ 1%). MSI testing should also be considered at baseline to guide management of the MSI-H subgroup, in whom tumors have been shown to be very sensitive to IO.

IO is highly effective in the second-line treatment for ESCC in patients with high PD-L1 (CPS ≥10) expression, offering OS and PFS benefit as well as statistically significantly higher response rates, and this was confirmed in our pooled analysis. Similarly, statistically significant survival benefit has been observed in patients with ESCC and CPS greater than or equal to 1, although it is still unclear if they respond better. The role if IO in the CPS less than 1 group, needs to be further investigated to ascertain the benefit in this group as well. Nevertheless, the efficacy of IO has not been proven in second-line upper GI adenocarcinoma or in maintenance treatment after disease control with first-line chemotherapy. Thoughtfully designed trials with the appropriate biomarker selection will be necessary to navigate the future management of patients with advanced upper GI malignancies with the availability of IO, chemotherapy, and targeted therapies in the repertoire of treatment options available.

## Funding

None.

## Notes

**Role of the funder:** Not applicable.

**Disclosures:** All authors disclose any conflict of interest.

**Author contributions:** Conceptualization: KK, DM; Investigation: AA, DM, GZ, IG, KK, TG, MN, MT; Data Curation: DM, KK, PN; Formal analysis: DM, KD, PN; Writing-original draft: KK, DM, MT; Writing-review and editing: AA, GZ, IG, KD, MN, PN, TG

## Data Availability

The data underlying this article are available in the article and in its online supplementary material.

## Supplementary Material

pkab088_Supplementary_Data
