# Supplementary material for: Immunotherapy Efficacy in the Initial Lines of Treatment in Advanced Upper Gastrointestinal Malignancies: A Systematic Review of the Literature
Source: JNCI Cancer Spectr. 2021 Nov 16;5(6):pkab088. doi: 10.1093/jncics/pkab088 (PMC8677514; doi:10.1093/jncics/pkab088)

SUPPLEMENTARY MATERIALS

Systematic review methodology

Search strategy

A literature search in three medical libraries was performed by two independent reviewers: Pubmed (AA, DM), Embase (TG, MN), and the Cochrane library (DM, TG) for relevant reports using the following algorithm: (avelumab OR atezolizumab OR nivolumab OR pembrolizumab OR durvalumab OR ipilimumab OR cemiplimab OR immunotherapy) AND (gastric OR stomach OR esophageal OR esophagogastric OR gastroesophageal) AND (cancer OR neoplasm OR carcinoma OR adenocarcinoma). (Last search: November 2020). In case of disagreement between independent searches KK (gastrointestinal oncology expert) supervised the data.
To ensure that electronic searches would not miss reports of eligible studies, two independent reviewers also searched for the years 2016 through 2020 several oncology journals that publish randomized trials: *New England Journal of medicine, Lancet, Lancet Oncology, Journal of Clinical Oncology*, JAMA Oncology and *Annals of Oncology(MT, KK)* [1].
The reference list of retrieved papers was further screened for additional publications to minimize publication bias (MN, IG).
Furthermore, as recent trials may still be unpublished, we also reviewed abstract books and presentations of major recent meetings in 2016 till June 2020 of the American Society of Clinical Oncology, the European Society of Clinical Oncology, to identify any other trial that had presented final data and comparisons that would be eligible for consideration in the meta-analysis (American Society of Clinical Oncology ASCO, European Society of Medical Oncology ESMO, European Cancer Organization ECCO annual meeting, ESMO World Congress on Gastrointestinal Cancer and ASCO Gastrointestinal Cancer Symposium) (GZ, IG). Earlier meeting abstracts were not included. Abstracts presented in the ASCO 2021 congresses were included in the survival analysis to provide the most up-to date evidence.

Eligibility Criteria

To reach the best level of solid available evidence, we considered as eligible only Randomized Control Trials (only randomized studies) that compare the use of check point inhibitors versus chemotherapy for the treatment of patients with advanced/metastatic gastric, esophageal and esophago-gastric cancer either as immunotherapy alone (IO) and IO combined to chemotherapy. To be eligible studies should include only adenocarcinoma and/or squamous cell carcinoma histology.
Studies used in the analysis included trials in 1st line, 2nd line and maintenance treatment after response to 1st line treatment. Trials were eligible regardless of the doses and schedules used for the regimens compared.
Whenever multiple reports pertained to overlapping groups of patients, for the meta-analysis of cumulative available randomized evidence, we retained only the report with longest follow-up (largest number of events) for calculations, to avoid duplication of information. Data from interim analyses and abstracts reports were eligible if no further final data were available. To be included in the meta-analyses of cumulative available randomized evidence, studies should have been evaluating the therapeutic contribution of immunotherapy in patients with upper gastrointestinal tract malignancies (i.e. esophageal, gastric, or gastroesophageal junction cancers). We excluded single-arm studies, dose-escalation studies, and non-randomized and pseudo-randomized trials (e.g., those with alternate allocation of subjects).

Data extraction

From each eligible trial (DM, KK, NP) we recorded the following items for both arms: authors' names; journal and year of publication; country of origin; years of patient enrolment; number of centers involved; number of patients randomly assigned and analyzed per arm, age, site of metastases, the exact regimens used and their dose and schedule; the line of treatment; and any additional treatments given to both arms.
We recorded study design items, including whether there was a description of the mode of randomization, allocation concealment, the number of withdrawals per arm and blinding [2] and whether any planned or unplanned interim analyses had been performed [3]. We also recorded the median survival by arm and whether any statistically significant difference had been detected between the compared arms at a p value of 0.05.

Flow of the systematic review and eligible reports identified

Overall algorithmic searches of medical libraries retrieved 8851 hits (EMBASE N=5587, PUBMED/MEDLINE N= 2728, and the COCHRANE LIBRARY N=536), pertaining eligible trials. [4–12]. Hand searches of major related conferences and medical journals permit to identify 8 more studies to be included in the review [13–20]. Of these, six were pertaining 1st line randomized studies [4,13,15–

18], eight second line randomized studies [5–9,12,19,20], and three maintenance randomized trials [10,11,14].Two studies were not included in the pooled analysis as immunotherapy was not compared with chemotherapy [12,21]. The flow and the result of the systematic review of literature for eligible trials are reported in the flow chart below (supplementary figure 1).

Name list for the systematic review of the literature: AA (Anna-Lea Amylidi), DM (Davide Mauri), GZ (George Zarkavelis), IG (Ioanna Gazouli), KK (Konstantinos Kamposioras), MN (Michail Nikolaou) MT (Maria Tolia), TG (Theodora Germetaki).

First line treatment

Calculation of cumulative available randomized evidence (Pooled Analysis) for the use of immunotherapy compared to standard chemotherapy

a) Descriptive analysis

The systematic review of literature identified overall four randomized trials comparing the use of immunotherapy vs. standard chemotherapy as first line regimen in Upper Gastrointestinal Tract Malignancies. All four trials compared chemo-immunotherapy vs. standard chemotherapy [4,13,15,16]. Data presented at ASCO 2021 identified two more 1st line trials with survival analyses [17,18]. In one study immunotherapy single agent was compared with standard chemotherapy [4]. Thereafter the summary analysis was performed only for the six studies comparing the combination IO-chemo vs. chemotherapy. All trials analyzed were multicenter [4,13,15–18].

For the **KEYNOTE 062** (NCT02494583) a randomized phase III, controlled, partially blinded trial, patients were randomly allocated (1:1:1) via a central interactive voice response and integrated web response system (Almanac Clinical Technologies) to pembrolizumab (200mg every 3 weeks), pembrolizumab plus chemotherapy and placebo plus chemotherapy [4]. The chemotherapy regimen used in the study was cisplatin 80 mg/m^2 on day 1 plus fluorouracil 800 mg/m^2 on days 1-5 or capecitabine 1000 mg/m^2 twice daily on days 1-14 every 3 weeks [4]. Primary outcome of the study was Overall Survival (OS) evaluation in patients with PD-L1 CPS 1 or greater (intention-to-treat population), and PD-L1CPS 10 or greater, and progression free survival (PFS) per Recist 1:1 by blinded independent central review (BCIR) in PD-L1 CPS 1 or greater [4]. Study was global recruiting patients from Australia, Asia, North- Central- and South-America and Europe, (from 200 centers in 29 countries) and allocating patients to each treatment by block of 6 per stratum. Overall 1787 patients were screened, and 763 were randomized in the trial between September, 2015, and May, 2017[4], Nonetheless 3 patients randomized in the pembrolizumab arm, 6 patients in the chemotherapy arm and 6 patients in the chemo plus pembrolizumab arm did not receive the allocated treatment, thus only 748/763 patients received the allocated regimen [4]. Since in the KEYNOTE 062 study the survival outcome used a 99.2% CI level, in our report all the reported outcomes were recalculated at CI level of 95%. Of note in KEYNOTE 062 study only patients with PD-L1 CPS of 1 or greater were included in the study [4].

For the **ATTRACTION-4**/ ONO-4538-37(NCT02746796), a randomized phase II/III two-part study trial, safety and efficacy data of the phase II study were officially published in 2019 [22], while preliminary survival outcomes of the second part (phase III) of the study were presented at 2020 ESMO meeting [13]. In the phase III trial 724 patients were recruited from 130 centers in three Asian countries (Japan, Korea, and Taiwan) between March 2017 and May 2018 [13]. Patients were randomized 1:1 using a centralized interactive web response system [22]. The study investigated whether the use of Nivolumab 360mg iv /3wks plus chemotherapy may have survival advantages when compared to chemotherapy plus placebo (chemotherapy alone). The chemotherapy regimen used in both experimental and control arm was oxaliplatin 130 mg/m2 plus Tegafur-gimeracil-oteracil 40 mg/ m2 bid or Capecitabine 1000 mg/ m2 bid orally (days 1-14), followed by 7 days off, every three weeks. Co-primary endpoints were PFS (central assessment by an independent review committee) and OS [13]. Two patients randomized in the experimental arm and three patients in the control arm did not receive the allocated treatment, thus 717/724 patients received the allocated regimen; nonetheless analyses were performed in the intention-to-treat population [13].

For the **KEYNOTE 590** (NCT03189719), a randomized, double-blind, placebo-controlled phase III clinical trial of pembrolizumab 200mg (MK-3475) in combination with cisplatin 80 mg/m^2 on day 1 plus fluorouracil 800 mg/m^2 on day 1-5 every three weeks *vs.* placebo plus the same chemotherapy regimen (cisplatin 80 mg/m^2 on day 1 plus fluorouracil 800 mg/m^2 on day 1-5), a randomization 1:1 by parallel assignment was performed. The exact randomization modality is actually not described in formal sources [16,23], as the study is still formally unpublished and the only data available from this study are from trial presentation at last 2020 ESMO conference and ClinicalTrials.gov. Overall 749 patients were recruited from 189 centers globally between July 2017 to June 2019 [16,23], 373 assigned to the investigational arm and 376 placebo arm; of these 370 patients received the assigned treatment in each arm (740/749) [16]. Dual-primary endpoints were OS and PFS: overall survival in participants with esophageal squamous cell carcinoma (ESCC) whose tumors PD-L1CPS ≥10; OS in participants with ESCC;OS in participants whose tumors PD-L1CPS ≥10; OS in all participants, PFS per RECIST 1.1 in ESCC patients, PFS in participants whose tumors PD-L1CPS ≥10,andPFS in all participants [6]. Survival outcomes were analyzed in intention-to-treat (ITT) method [16].

For the **CHEKMATE 649** (NCT02872116) trial, a randomized, open-label phase 3 study, initially scheduled as three arm study, patients were randomized between combinational immunotherapy (nivolumab 1 mg/kg + ipilimumab 3mg/kg every three weeks for 4 weeks and then nivolumab 240mg every two weeks) vs. combinational chemo-immunotherapy (nivolumab 360mg plus XELOX every three weeks *OR* nivolumab 240mg plus FOLFOX every two weeks) *vs.* chemotherapy alone (XELOX every three weeks *OR* FOLFOX every two weeks) [15]. XELOX regimen comprised the use of oxaliplatin 130 mg/m^2 plus capecitabine 1000 mg/ m^2 bid orally days 1-14, while FOLFOX regimen was oxaliplatin85 mg/m^2, leucovorin 400 mg/m^2, 5-FU 400 mg/m^{2}and continual infusion 5-FU 1200 mg/m^2 days 1-

2 [15]. Nonetheless the NIVO1-IPI3 combinational immunotherapy arm was closed before patients enrolled and the study continue as phase III, two arms RCT, randomizing in 1:1 parallel assignment [24]. The exact randomization modality was actually not described in formal sources [15,24] at the time of this review, and the only data available from this study are from the trial presentation at last 2020 ESMO conference and ClinicalTrials.gov [15,24] .Overall 1581 patients were recruited from 176 centers globally since October 2016 [15,24], *782 of those (out of 789 allocated) received the experimental chemo-immunotherapy* %) in the control arm did not receive the allocated treatment [24], nonetheless survivals outcomes were analyzed in ITT [15]. It should be noted that the 60% of the patients enrolled in the study (955/1581) were with PD-L1 CPS equal or greater than 5 [15].
For the **CHEKMATE 648** (NCT03143153) trial, a randomized, open-label phase 3 study, three arm study, patients were randomized between combinational immunotherapy (nivolumab 3 mg/kg every two weeks + ipilimumab 1mg/kg every six weeks) vs. combinational chemo-immunotherapy with nivolumab 240mg every two weeks plus chemo (fluorouracil + cisplatin every four weeks) *vs.* chemotherapy alone as the first-line treatment for advanced esophagal squamous cell carcinoma (ESCC). Randomization with parallel assignment was performed, nonetheless, the exact randomization modality is actually not described in formal sources [17,25] as the study is still formally unpublished and the only data available from this study are from the trial presentation at last 2021 ASCO conference and ClinicalTrials.gov [17,25] . Overall 970 patients were recruited from 27 countries globally from 29th June 2017 [17,25]. *321 received the experimental chemo-immunotherapy treatment* and 324, chemotherapy only. Survival outcomes were analyzed in ITT [17]. The 49% of the patients enrolled in the study were with PD-L1 CPS equal or greater than 1 [17].

For the **ESCORT-1st** (NCT03691090) trial [18,26], a randomized double-blind, placebo-controlled 1:1, phase 3, two arms study. Patients were randomized to receive camrelizumab 200 mg or placebo, both combined with up to 6 cycles of paclitaxel (175 mg/m^2) and cisplatin (75 mg/m^2). All were given intravenously every three weeks as the first-line treatment for advanced esophagal squamous cell carcinoma (ESCC). The exact randomization modality is actually not described in formal sources [18,26], as the study is still formally unpublished and the only data available from this study are from the trial presentation at last 2021 ASCO conference and ClinicalTrials.gov [18,26]. Overall 596 patients were recruited from Chinese Hospitals from the 3rd of December 2018 to the 12th May 2020, *298 of those received the experimental chemo-immunotherapy treatment* and 297chemotherapy only [18]. Survival outcomes were analyzed in ITT. The 49% of the patients enrolled in the study were with PD-L1 CPS equal or greater than 1 [18].
.

b) **Studies overview:**

Although the model of randomization was described in two of the six analyzed studies [4,22], withdrawals were described in the five studies [4,13,15–17]. All studies were randomized phase III trials, including OS and PFS in their primary outcomes, and relative analyses were performed in ITT [4,13,15–18]. Only one study enrolled patients with PD-L1 CPS of one or greater [4]. Thereafter data for the overall patients population (independently of PD-L1 CPS expression) are available only for 5 trials [13,15–18]. In the interventional arm, two studies used pembrolizumab [4,16], three nivolumab [13,15,17] and one camrelizumab [18]. In five studies the chemotherapy regimen used included a platinum compound (cisplatin or oxaliplatin) plus a fluoropyrimidine agent (5-FU, capecitabine, tegafur-gimeracil-oteracil) [4,13,15–18]. One study used paclitaxel plus cisplatin as the chemotherapy regimen [18]. No anthracyclines were used [4,13,15–18].
Overall 5363 were enrolled and randomized across six trials, of those 4801 patients were eligible for cumulative analysis as they were randomized to combined chemo-immunotherapy with checkpoint inhibitors (n = 2400) vs. chemotherapy alone (n=2401). Overall 61 patients did not receive allocated treatment, 19 in experimental arms and 42 in controls arms. Since survival outcomes (OS and PFS) were analyzed in the intention-to-treat method, 4802 patients were eligible for analysis of the primary outcomes (2400 in the chemo-immunotherapy arm and 2402 in the standard chemotherapy arm).
Primary outcome of the pooled analyses was to assess the cumulative actually available randomized evidence for overall survival and progression free survival for the use of 1st line immunotherapy in the treatment of esophago-gastric cancer when compared to standard 1st line chemotherapy. Secondary outcome was to assess whether the stratification by PDL1 status, histology and tumor location may have any effect on the calculated comprehensive randomized evidence for overall survival and progression free survival. Comprehensive analysis for response rate was also part of the secondary outcome.

Maintenance treatment:

Calculation of cumulative available randomized evidence (Pooled Analysis) for the use of maintenance immunotherapy compared to no maintenance

a) **Descriptive analyses**

The systematic review of literature, identified overall two randomized trials comparing the use of maintenance immunotherapy vs no maintenance IO in Upper Gastrointestinal Tract Malignancies [10,11].

Data presented at ASCO 2021 identified one more maintenance trial with PFS analysis [14].

The first trial (NCT01585987), was a phase II open –label randomized trial, evaluating the use of an *anti-CTLA4* antibody immunotherapy, ipilimumab (investigational arm) versus best supportive care (BSC) as maintenance treatment among patients with advanced / metastatic gastric or gastroesophageal junction cancer who achieved at least stable disease with first line chemotherapy with a platinum plus fluoropyrimidine regimen [10]. Ipilimumab was administered as 10mg/kg every 3 weeks for four doses, then 10 mg/kg every 12 weeks for up to three years, while BSC could include also the continuation of fluoropyrimidine until progression or toxicity. Between July 2012 and July 2014 a total of 143 patients with gastric or gastroesophageal junction from 12 countries across ASIA, Europe and North America were enrolled; 114 of those were randomized by 1:1 assignment: 57 in the investigational arm and 57 in the control arm, however only 51/57 in the BSC arm received the assigned management. Cross over was not allowed. Database locks occurred on June 2014 due to trial cessation on a preplanned interim analysis. Primacy outcome was the evaluation of immune-related Progression Free Survival (irPFS) as per assessment of a blinded independent review committee (IRC) according to immune related response criteria (irRC). Overall survival analysis was part of secondary outcomes [10].

The JAVELIN Gastric 100 trial (NCT02625610), was an open-label, parallel assignment, multicenter, randomized phase III trial. All patients received the same first-line induction therapy for up to 12 weeks with one of three regimens: A) oxaliplatin 85 mg/m^2 intravenously (IV) and leucovorin 200 mg/m^2 (or equivalent levoleucovorin dose), followed by fluorouracil (FU) 2,600 mg/m^2 by continuous infusion over 24 hours on day 1, every 2 weeks; B) oxaliplatin 85 mg/m^2 IV and leucovorin 400 mg/m^2 (or equivalent levoleucovorin dose), followed by FU 400 mg/m^2 IV on day 1 and FU 2,400 mg/m^2 by continuous infusion over 46 to 48 hours on days 1 to 2, every 2 weeks; C) or oxaliplatin 130 mg/m^2 IV on day 1 and capecitabine 1,000 mg/m^2 orally twice daily for 2 weeks, followed by a 1-week rest period, every 3 weeks [11]. Patients without progressive disease (PD) per RECIST (version 1.1) after induction chemotherapy, confirmed by an independent radiologist, were randomly assigned 1:1 to either maintenance therapy with avelumab 10 mg/kg IV every 2 weeks or continuation of the same chemotherapy. All patients received best supportive care (BSC). The primary end point was OS (time from random assignment to death resulting from any cause). OS was assessed in all randomly assigned patients and in randomly assigned patients with PD-L1–positive tumors (PD-L1 protein expression in $\geq$ 1% of tumor cells). PD-L1 status was assessed centrally at baseline using the PD-L1 immunohistochemical (IHC) 73-10 performance evaluation–only assay (Agilent Technologies/Dako, Carpinteria, CA). Study was global recruiting patients from Australia, Asia, North America and Europe (178 sites in 17 countries). Between December 31-2015, and November 29 - 2017, 805 patients were enrolled, 499 of those achieved disease control in the 12-week induction phase and were randomly assigned to Avelumab maintenance (n = 249) or continue chemotherapy (n = 250). Fifty four patients (30 in the interventional arm and 24 in the chemotherapy arm) were PD-L1–positive (expression in $\geq$ 1% of tumor cells). In the chemotherapy arm, seven patients were considered unsuitable for further chemotherapy and received BSC only [11].

The PLATFORM (NCT02678182) is a prospective, open-label, multicentre, adaptive phase II , 5-arm trial assessing maintenance therapy in patients with esophago-gastric adenocarcinoma after platinum-based first-line induction chemotherapy. HER2 negative patient were initially randomized 1:1:1:1:1 to surveillance (arm 1), capecitabine (arm 2), durvalumab (arm 3), with rucaparib (arm 4) and capecitabine + ramucirumab (arm 5) [14]. Arm 3 (Durvalumab10mg/kg iv, every two weeks) prematurely closed due to cessation of industry support. At data lock on 2nd February 2021, a total of 205 patients were concurrently randomized into arm1 (n = 100) and arm 3 (n = 105), overall survival and progression free survival data were recently presented at ASCO 2021 annual meeting [14].

b) **Studies overview:**

Overall 1010 patients were enrolled in the two maintenance trials, and 818 patients were randomized in maintenance line trials and eligible for intention-to-treat analysis (ITT analysis), 411 in the immunotherapy arm and 407 in the standard maintenance arm.

All identified trials analyzed only adenocarcinoma histology and described withdrawals in detail while no study described in detail the randomization modality. [10,11,14,27,28]. All trials analyzed were multicenter [10,11,14].The first study was a phase II trial immune-related progression free survival (irPFS) as primary outcome [10]], while the second study was a phase III study with primary outcome the evaluation of overall survival as in all randomly assigned patients and in those with PD-L1–positive tumors [11]. The third study was an adaptive phase II 5 arm trial [14].

Of note, in the first study an *anti-CTLA4* antibody (ipilimumab) was used as checkpoint inhibitor [10], while in the second and third study an anti PD-L1 antibodies (avelumab and durvalumab respectively) was used [11,14]. Thereafter cumulative results in this sub-setting are not stringent, pertains only the overall general involvement of cheek-point inhibitors molecules in the maintenance treatment of upper GI tumor, but did not give additional information on the performance of each targetable checkpoint-pathway. No taxanes or anthracyclines was used in first line chemotherapy regimens. No study used atezolizumab, nivolumab, or pembrolizumab as a maintenance treatment in the investigational arm [10,11,14].

Primary outcome of the pooled analyses was to assess the cumulative actually available randomized evidence for overall survival and progression free survival for the use of maintenance check point inhibitor immunotherapy in the treatment of esophago-gastric cancer when compared to standard chemotherapy alone. Stratification by histology was not possible since from inclusion criteria all patients should have had adenocarcinoma histology. Comprehensive analyses for additional response rate were part of our secondary outcomes. Since we are dealing with maintenance studies, we define additional responses the additional objective responses achieved after the randomization for the maintenance treatment after the first line regimen (induction regimen).

Second line treatment:

Calculation of cumulative available randomized evidence (Pooled Analysis) for the use of immunotherapy compared to standard chemotherapy

c) Descriptive analysis

The systematic review of literature identified overall five randomized trials comparing the use of immunotherapy vs. standard chemotherapy in second line management of Upper Gastrointestinal Tract carcinomas [5–8,19]. Data presented at ASCO 2021 congress identified one more trial [20].

The **ESCORT trial** (**NCT03099382)** was an open label phase III randomized trial. Patients with advanced/metastatic esophageal squamous cell carcinoma were recruited from 43 hospitals in China between May 2017 to July, 2018 [8], and randomized to receive either camrelizumab200mg (every two weeks OR the investigators' choice chemotherapy (Docetaxel 75 mg/m² on day 1 of each 3-week cycle, OR irinotecan 180 mg/m², on day 1 of each 2-week cycle) as second line treatment for the disease. Randomization assigned (1:1) was done using the Randomization and Trial Supply Management system (RTSM system; Bioknow, Beijing, China) via central block randomization method. The randomization sequences were generated by a randomization specialist of the sponsor in SAS (version 9.4). The sponsor's study team was masked to treatment allocation. The primary endpoint was overall survival. In total 457 patients were randomized, 229 in the experimental arm and 228 in the control arm, nonetheless only 228 and 220 received the allocated regimens (6 consent withdrawn, 2 inclusion criteria violation, 1 other reason) [8].

The **ATTRACTION-3trial (NCT02569242)** was an open-label randomized phase 3 trial. Patients were recruited from 90centers in Asia, Europe and North America (Denmark, Germany, Italy, Japan, South Korea, Taiwan, the UK, and the USA) [6]. Between January 2016, and May 2017,419 patients affected by advanced esophageal squamous cell carcinoma were randomly (1:1) assigned to receive second line treatment either with nivolumab 240 mg every 2 weeks (n =210), or investigator's choice of chemotherapy (paclitaxel 100 mg/m2 once per week for 6 weeks followed by 1 week off OR docetaxel 75 mg/m2 every 3 weeks) n=209. Of those 1 patients per arm did not receive the allocated regimen because of death (investigational arm) and decision withdrawal (chemotherapy arm) [6]. Randomization was done using an interactive web response system with a block size of four; an authorized vendor generate the sequentially numbered containers to ensure random allocation, and to assign patients to study treatments; and the web registration system ensured that the container sequence was concealed until the treatment allocation was completed. The primary endpoint was overall survival [6]..

The KEYNOTE181 trial (NCT03933449), was a global, open-label randomized phase 3 trial. Patients with advanced or metastatic esophageal cancer that progressed after one line of prior therapy were recruited from154 centers in 32 countries between December 2015 and June 2017 [5]. *Both squamous and adenocarcinoma histology were allowed.*

Overall, 628 patients were randomly assigned 1:1 to pembrolizumab 200 mg every 3 weeks *vs.* investigator's choice of standard of care chemotherapy (paclitaxel 80-100 mg/m2 on days 1, 8, and 15 of each 28-day cycle, OR docetaxel 75 mg/m2 on day 1 of each 21-day cycle, OR irinotecan 180 mg/m2 on day 1 of each 14-day cycle [5]. Among those 314 patients were randomized to the pembrolizumab arm and 314 to chemotherapy arm; 4 patients in the chemotherapy arm did not receive the allocated treatment. and The study had three primary end points: overall survival in patients with PD-L1 CPS $\geq$10, in patients with squamous cell carcinoma, and in all patients.

The KEYNOTE 061 trial (NCT02370498), was a global randomized, open-label, phase 3 study. Patients with advanced gastric or gastroesophageal junction cancer that progressed after one line of prior therapy were recruited from 148 centers in 30 countries between June 2015 and July 2016 [7]. *Only adenocarcinoma histology was allowed.* Overall, 592 patients were randomly assigned 1:1 using a central interactive voice-response and integrated web-response system to receive pembrolizumab 200 mg every 3 weeks (n = 296) or to paclitaxel 80 mg/m2 intravenously on days 1, 8, and 15 of 4-week cycles (n = 296).Of those only 294 / 296 in the experimental arm and 276/296 received the allocated treatment. Reasons of not receiving allocated treatment were not described. The allocation schedule was generated by the system vendor using a computerized random list generator. Primary endpoints were overall survival in patients with a PD-L1 CPS of 1 or higher.

The KEYNOTE 063 trial (NCT03019588) was an Asian, multicenter, open-label randomized phase 3 trial; patients with advanced gastric or gastro-esophageal junction adenocarcinoma that progressed after one line of prior therapy should have been recruited from 36 centers in China, Korea, Taiwan and Malaysia from February 2017 Nonetheless, the study was prematurely stopped after the presentation of the results from the KEYNOTE-061 where not survival benefit of pembrolizumab vs. paclitaxel was demonstrated [7], Overall 94 patients were recruited and 1:1randomized to receive pembrolizumab 200 mg every 3 weeks (n = 47) or to standard dose

paclitaxel [19]. Primary endpoints were both overall survival and PFS [29]. Only data from an abstract presentation to ASCO 2020 were available for this study [19].

The RATIONALE 302 trial (NCT03430843) [20] is a global randomized phase 3 study involving 132 sites in 10 countries. Adults with histologically confirmed advanced/unresectable or metastatic ESCC whose disease progressed following prior systemic therapy, entered the study [20,30]. Overall, 512 patients were 1:1 randomized to receive Tislelizumab 200 mg intravenously every 3 weeks (n=256) or investigator-chosen standard chemotherapy - paclitaxel, docetaxel, or irinotecan- (n=256) and treated until disease progression, unacceptable toxicity, or withdrawal. Primary endpoints was overall survival in all randomized patients [20]. Only data from an abstract presentation to ASCO 2021 were available for this study [20,30]

d) **Studies overview:**

All six trials analyzed were multicenter phase 3 randomized controlled trials, including OS in their primary outcomes, and relative analysis was performed with intention-to-treat (ITT) method [5–8,19,20]. Four of these were global [5–7,20], one Asian [19] and one was a national Chinese study [8]. All studies described the mode of randomization [5–8,19,20], nonetheless the exact randomization process was correctly described only three trials [6–8]. Accordingly, the role of funding sources was described in all completed trials in their full text publication [5–8], but not in the Asian trials that was prematurely interrupted and for which we have only an abstract presentation at last ASCO and some details from the NCT03019588 registration [19]. Correct withdrawals description was available only in two trials [6,8]. In one trial, 7% of the patients in the chemotherapy arm did not receive the allocated treatment [7]. Quality of life assessment part of the study outcomes only in two studies [6,8]. All six trials compared IO monotherapy vs. standard chemotherapy as second line treatment [5–8,19,20]. Four studies included patients with esophageal carcinomas [5,6,8]; three of them only with squamous histology [6,8]. The other two trials enrolled patients with G/GEJ adenocarcinomas [7,19]. In the interventional arms, one study used Camrelizumab [8], one Nivolumab [6], one Tislelizumab [20] and 3 Pembrolizumab [5,7,19] while as chemotherapy comparator a taxane based regimen (paclitaxel or docetaxel) was used in 5 studies [5–8,19], irinotecan based regimen was feasible in three trials [5,8,20].

Overall 2702 were enrolled and randomized across five trials and were eligible for cumulative analyzes since randomized to immunotherapy with checkpoint inhibitors (n = 1352) vs. chemotherapy alone (n=1350). Of those, 2651 were treated with the allocated regimens: 1348 (99.7%) in the experimental arms and 1303 (96.5%) in the control arms. Probability for receiving the treatment was statistically lower in the control arm (OR 12.1558, 95% CI: 4.3672 - 33.8349, z = 4.782, p < 0001). Overall 51 patients did not received allocated treatment, 4 in experimental arms and 47 in controls arms. Since survival outcomes (OS and PFS) were analyzed in ITT methodology, 2702 patients were eligible for analyses of our primary outcomes; 1348 in the immunotherapy arms and 1303 in the standard chemotherapy arms.

Primary outcome of the pooled analyses was to assess the cumulative actually available randomized evidence for overall survival and progression free survival for the use of 2st line immunotherapy in the treatment of esophago-gastric cancer when compared to standard 2st line chemotherapy. Secondary outcome was to assess whether the stratification by PD-L1 status, histology and MSI may have any effect on the calculated comprehensive randomized evidence for overall survival and progression free survival. Comprehensive analyses for response rate were also part of our secondary outcome.

References

[1] Hopewell S, Clarke M, Lusher A, Lefebvre C, Westby M. A comparison of handsearching versus MEDLINE searching to identify reports of randomized controlled trials. Stat Med 2002;21:1625–34. https://doi.org/10.1002/sim.1191.

[2] Higgins JPT, Thomas J, Chandler J, Cumpston M, Li T, Page MJ, Welch VA (editors). Cochrane Handbook for Systematic Reviews of Interventions version 6.2 (updated February 2021). Cochrane, 2021. Available from www.training.cochrane.org/handbook. n.d.

[3] Montori VM, Devereaux PJ, Adhikari NKJ, Burns KEA, Eggert CH, Briel M, et al. Randomized Trials Stopped Early for Benefit. JAMA 2005;294:2203. https://doi.org/10.1001/jama.294.17.2203.

[4] Shitara K, Van Cutsem E, Bang Y-J, Fuchs C, Wyrwicz L, Lee K-W, et al. Efficacy and Safety of Pembrolizumab or Pembrolizumab Plus Chemotherapy vs Chemotherapy Alone for Patients With First-line, Advanced Gastric Cancer. JAMA Oncol 2020;6:1571. https://doi.org/10.1001/jamaoncol.2020.3370.

[5] Kojima T, Shah MA, Muro K, Francois E, Adenis A, Hsu C-H, et al. Randomized Phase III KEYNOTE-181 Study of Pembrolizumab Versus Chemotherapy in Advanced Esophageal Cancer. J Clin Oncol 2020;38:JCO.20.01888. https://doi.org/10.1200/jco.20.01888.

[6] Kato K, Cho BC, Takahashi M, Okada M, Lin CY, Chin K, et al. Nivolumab versus chemotherapy in patients with advanced oesophageal squamous cell carcinoma refractory or intolerant to previous chemotherapy (ATTRACTION-3): a multicentre, randomised, open-label, phase 3 trial. Lancet Oncol 2019;20:1506–17. https://doi.org/10.1016/S1470-2045(19)30626-6.

[7] Shitara K, Özgüroğlu M, Bang YJ, Di Bartolomeo M, Mandalà M, Ryu MH, et al. Pembrolizumab versus paclitaxel for previously treated, advanced gastric or gastro-oesophageal junction cancer (KEYNOTE-061): a randomised, open-label, controlled, phase 3 trial. Lancet 2018;392:123–33. https://doi.org/10.1016/S0140-6736(18)31257-1.

[8] Huang J, Xu J, Chen Y, Zhuang W, Zhang Y, Chen Z, et al. Camrelizumab versus investigator's choice of chemotherapy as second-line therapy for advanced or metastatic oesophageal squamous cell carcinoma (ESCORT): a multicentre, randomised, open-label, phase 3 study. Lancet Oncol 2020;21:832–42. https://doi.org/10.1016/S1470-2045(20)30110-8.

[9] Janjigian YY, Bendell J, Calvo E, Kim JW, Ascierto PA, Sharma P, et al. CheckMate-032 Study: Efficacy and Safety of Nivolumab and Nivolumab Plus Ipilimumab in Patients With Metastatic Esophagogastric Cancer. J Clin Oncol 2018;36:2836–44. https://doi.org/10.1200/JCO.2017.76.6212.

[10] Bang YJ, Cho JY, Kim YH, Kim JW, Bartolomeo M Di, Ajani JA, et al. Efficacy of sequential ipilimumab monotherapy versus best supportive care for unresectable locally advanced/metastatic gastric or gastroesophageal junction cancer. Clin Cancer Res 2017;23:5671–8. https://doi.org/10.1158/1078-0432.CCR-17-0025.

[11] Moehler M, Dvorkin M, Boku N, Özgüroğlu M, Ryu M-H, Muntean AS, et al. Phase III Trial of Avelumab Maintenance After First-Line Induction Chemotherapy Versus Continuation of Chemotherapy in Patients With Gastric Cancers: Results From JAVELIN Gastric 100. J Clin Oncol 2020:JCO.20.00892. https://doi.org/10.1200/jco.20.00892.

[12] Kelly RJ, Lee J, Bang Y-J, Almhanna K, Blum Murphy M, Catenacci DVT, et al. Safety and Efficacy of Durvalumab and Tremelimumab Alone or in Combination in Patients with Advanced Gastric and Gastroesophageal Junction Adenocarcinoma. Clin Cancer Res 2019:clincanres.2443.2019. https://doi.org/10.1158/1078-0432.CCR-19-2443.

[13] Boku N, Ryu MH, Oh D-Y, Oh SC, Chung HC, Lee K-W, et al. LBA7_PR Nivolumab plus chemotherapy versus chemotherapy alone in patients with previously untreated advanced or recurrent gastric/gastroesophageal junction (G/GEJ) cancer: ATTRACTION-4 (ONO-4538-37) study. Ann Oncol 2020;31:S1192. https://doi.org/10.1016/j.annonc.2020.08.2297.

[14] Fong C, Patel B, Peckitt C, Bourmpaki E, von Loga K, Begum R, et al. Maintenance durvalumab after first-line platinum-based chemotherapy in advanced oesophago-gastric (OG) adenocarcinoma: Results from the PLATFORM trial. J Clin Oncol 2021;39:4015–4015. https://doi.org/10.1200/JCO.2021.39.15_suppl.4015.

[15] Moehler M, Shitara K, Garrido M, Salman P, Shen L, Wyrwicz L, et al. LBA6_PR Nivolumab (nivo) plus chemotherapy (chemo) versus chemo as first-line (1L) treatment for advanced gastric cancer/gastroesophageal junction cancer (GC/GEJC)/esophageal adenocarcinoma (EAC): First results of the CheckMate 649 study. Ann Oncol 2020;31:S1191. https://doi.org/10.1016/j.annonc.2020.08.2296.

[16] Kato K, Sun J-M, Shah MA, Enzinger PC, Adenis A, Doi T, et al. LBA8_PR Pembrolizumab plus chemotherapy versus chemotherapy as first-line therapy in patients with advanced esophageal cancer: The phase 3 KEYNOTE-590 study. Ann Oncol 2020;31:S1192–3. https://doi.org/10.1016/j.annonc.2020.08.2298.

[17] Chau I, Doki Y, Ajani JA, Xu J, Wyrwicz L, Motoyama S, et al. Nivolumab (NIVO) plus ipilimumab (IPI) or NIVO plus chemotherapy (chemo) versus chemo as first-line (1L) treatment for advanced esophageal squamous cell carcinoma (ESCC): First results of the CheckMate 648 study. J Clin Oncol 2021;39:LBA4001–LBA4001. https://doi.org/10.1200/JCO.2021.39.15_suppl.LBA4001.

[18] Xu R, Luo H, Lu J, Bai Y, Mao T, Wang J, et al. ESCORT-1st: A randomized, double-blind, placebo-controlled, phase 3 trial of camrelizumab plus chemotherapy versus chemotherapy in patients with untreated advanced or metastatic esophageal squamous cell carcinoma (ESCC). J Clin Oncol 2021;39:4000–4000. https://doi.org/10.1200/JCO.2021.39.15_suppl.4000.

[19] Chung HC, Kang Y-K, Chen Z, Bai Y, Wan Ishak WZ, Shim BY, et al. Pembrolizumab vs paclitaxel as second-line treatment for Asian patients with PD-L1–positive advanced gastric or gastroesophageal cancer (GC) in the phase III KEYNOTE-063 trial. J Clin Oncol 2020;38:e16586–e16586. https://doi.org/10.1200/JCO.2020.38.15_suppl.e16586.

[20] Shen L, Kato K, Kim S-B, Ajani JA, Zhao K, He Z, et al. RATIONALE 302: Randomized, phase 3 study of tislelizumab

versus chemotherapy as second-line treatment for advanced unresectable/metastatic esophageal squamous cell carcinoma. J Clin Oncol 2021;39:4012–4012. https://doi.org/10.1200/JCO.2021.39.15_suppl.4012.
[21] Janjigian YY, Bendell J, Calvo E, Kim JW, Ascierto PA, Sharma P, et al. CheckMate-032 Study: Efficacy and Safety of Nivolumab and Nivolumab Plus Ipilimumab in Patients With Metastatic Esophagogastric Cancer. J Clin Oncol 2018;36:2836–44. https://doi.org/10.1200/JCO.2017.76.6212.
[22] Boku N, Ryu M-H, Kato K, Chung HC, Minashi K, Lee K-W, et al. Safety and efficacy of nivolumab in combination with S-1/capecitabine plus oxaliplatin in patients with previously untreated, unresectable, advanced, or recurrent gastric/gastroesophageal junction cancer: interim results of a randomized, phase II trial (A. Ann Oncol 2019;30:250–8. https://doi.org/10.1093/annonc/mdy540.
[23] First-line Esophageal Carcinoma Study With Chemo vs. Chemo Plus Pembrolizumab (MK-3475-590/KEYNOTE-590) ClinicalTrials.gov https://clinicaltrials.gov/ct2/show/NCT03189719 Last accessed 31/12/2020 n.d.
[24] Efficacy Study of Nivolumab Plus Ipilimumab or Nivolumab Plus Chemotherapy Against Chemotherapy in Stomach Cancer or Stomach/Esophagus Junction Cancer (CheckMate649). Retrieved from: https://clinicaltrials.gov/ct2/show/NCT02872116. Last accessed 31/12/ n.d.
[25] Study to Evaluate Efficacy in Subjects With Esophageal Cancer Treated With Nivolumab and Ipilimumab or Nivolumab Combined With Fluorouracil Plus Cisplatin Versus Fluorouracil Plus Cisplatin (CheckMate 648) https://clinicaltrials.gov/ct2/show/NCT031431 n.d.
[26] Study of SHR-1210 in Combination With Chemotherapy in Advanced Esophageal Cancer. https://clinicaltrials.gov/ct2/show/NCT03691090 n.d.
[27] Planning Treatment for esophago-gastric Cancer: a Maintenance Therapy Trial (PLATFORM) https://clinicaltrials.gov/ct2/show/record/NCT02678182 n.d.
[28] An Efficacy Study in Gastric and Gastroesophageal Junction Cancer Comparing Ipilimumab Versus Standard of Care Immediately Following First Line Chemotherapy. NCT01585987. Retrieved from: https://clinicaltrials.gov/ct2/show/NCT01585987 n.d.
[29] Efficacy and Safety Study of Pembrolizumab (MK-3475) Versus Paclitaxel in Asian Participants With Advanced Gastric or Gastroesophageal Junction Adenocarcinoma Who Progressed After First-line Therapy With Platinum and Fluoropyrimidine (MK-3475-063/KEYNOTE- n.d.
[30] A Study of Tislelizumab (BGB-A317) Versus Chemotherapy as Second Line Treatment in Participants With Advanced Esophageal Squamous Cell Carcinoma https://clinicaltrials.gov/ct2/show/record/NCT03430843 n.d.

Supplementary Tables

Supplementary Table 1. Summary of Randomised trials in first line treatment (Overall Survival)[a]

Study	Line of Treatment	Tumour Site Histology	Arms	No. of patients	PD-L1 expression		
					Any	CPS≥1	CPS≥ 5[b] or 10[c]
					OS (95% CI), mos	OS (95% CI), ms	OS (95% CI), ms
KEYNOTE 062	1st	G/GEJ	Pembrolizumab	256	–	10.6 (7.7-13.8)	17.4 (9.1-23.1) [c]
		AdenoCa	Pl + Cis/5-Fu	250	--	11.1 (9.2-12.8)	10.8 (8.5-3.8)
						HR=0.91	HR = 0.69 (0.49-0.97)
						(99.2% CI = 0.69-1.18)	
KEYNOTE 062	1st	G/GEJ	Pembro/Chemo	257	--	12.5 (10.8-13.9)	12.3 (9.5-14.8) [c]
		AdenoCa	Pl + Cis/5-Fu	250	--	11.1(9.2-12.8)	10.8 (8.5-13.8)
						HR=0.85 (0.7-1.03)	HR = 0.85 (0.62-1.17)
ATTRACION-4	1st	G/GEJ	Nivo/Chemo	362	17.45 (15.67-20.83)	--	--
		AdenoCa	Pl + SOX/CapOx	362	17.15 (15.18-19.65)	--	--
					HR: 0.9 (0.75- 1.08)		
CheckMate-649	1st	G/GEJ/E	Nivo/Chemo	789	13.8 (12.6–14.6)	14.0 (12.6–15.0)	14.4 (13.1–16.2) [b]
		AdenoCa	Folfox/CapOx	782	11.6 (10.9–12.5)	11.3 (10.6–12.3)	11.1 (10.0–12.1)
					HR=0.8	HR = 0.77	HR = 0.71
					(99.3% CI = 0.68-0.94)	(99.3%CI = 0.64-0.92)	(98.4%CI = 0.59-0.86)
KEYNOTE-590	1st	E/GEJ Siewert I	Pembro/Chemo	373	12.4 (10.5-14.0)	--	13.5 (11.1-15.6) [c]
		AdenoCa/SCC	Pl+ Cis/5-Fu	376	9.8 (8.8-10.8)	--	9.4 (8.0-10.7)
					HR=0.73 (0.62 -0.86)		HR = 0.62 (0.49-0.78)
CheckMate-648	1st	Esophageal	Nivo/Chemo	321	13.2 (11.1-15.7)	15.4 (11.9-19.5)	--

NCT03143153		SCC	Cis/5-Fu	324	10.7 (9.4-11.9)	9.1 (7.7-10.0)	--
					HR= 0.74	HR = 0.54	
					(99.1% CI= 0.58-0.96)	(99.5% CI = 0.37-0.80)	
CheckMate-648	1st	Esophageal	Nivo/Ipi	325	12.8 (11.3-15.5)	13.7 (11.2-17.0)	--
NCT03143153		SCC	Cis/5-Fu	324	10.7 (9.4-11.9)	9.1 (7.7-10.0)	--
					HR: 0.78	HR = 0.64	
					(98.2% CI=0.62-0.98)	(98.6% CI = 0.46-0.90)	
ESCORT-1st	1st	Esophageal	Camrelizumab/Chemo	298	15.3 (12.8-17.3)	--	--
NCT03691090		SCC	Pac/Cis	298	12.0 (11.0-13.3)	--	--
					HR= 0.70 (0.56-0.88)		

[a] Adenoca = adenocarcinoma; Cap0x: capecitabine/oxaliplatin; Chemo: chemotherapy; CI: confidence interval; Cis: cisplatin; CPS = combined positive score; E: esophageal; FOLFOx: folinic acid + fluorouracil + oxaliplatin; G = gastric; GEJ = gastroesophageal junction; IPI: ipilimumab; HR: hazard ratio; ms: months; Nivo: nivolumab; OS= overall survival; Pac: paclitaxe; PD-L1: programmed cell death ligand-1; Pembro: pembrolizumab; Pl: placebo; SCC = squamous cell carcinoma; SOX = s1 (tegafur-gimeracil-oteracil potassium)/oxaliplatin; 5-Fu = 5-fluorouracil

[b] CPS expression ≥5%

[c] CPS expression ≥10%

Supplementary Table 2: Summary of Randomised trials in first line treatment (PFS, RR, DOR)

Study	Arms	PD-L1 expression			PD-L1 expression		PD-L1 expression	
		Any	CPS≥1	CPS≥ 5[b] or 10[c]	Any/CPS≥1[d]	CPS ≥ 5[b]/ 10[c]	Any/ CPS≥1[d]	CPS≥5[b]/10[c]
		PFS (95% CI), mos	PFS (95% CI), ms	PFS (95% CI), ms	RR % (n)	RR % (n)	DORms (95%CI)	DORms (95%CI)
KEYNOTE 062	Pembrolizumab	2.0 (1.5-2.8)	2.0 (1.5-2.8)	2.9 (1.6-5.4) [c]	14.8 (38/256)[d]	25 (23/92)[c]	13.7 (8.3-21.2)[d]	19.3 (8.3-NR)
	Pl + Cis/5-Fu	6.4 (5.7-7.0)	6.4 (5.7-7.0)	6.1 (5.3-6.9)	37.2 (93/250)[d]	37.8 (34/90)	6.8 (5.4- 8.2)[d]	6.8 (4.8-8.7)[c]
		HR=1.66 (1.37-2.01)	HR= 1.66 (1.37-2.01)	HR= 1.1 (0.79-1.51)				
KEYNOTE 062	Pembro/Chemo	–	6.9 (5.7-7.3)	--	48.6 (125/257)[d]	53 (52/99)[c]	6.8 (5.5- 8.3)[d]	8.3 (4.5-29.5)[c]
	Pl + Cis/5-Fu	--	6.4 (5.7-7.0)	--	37.2 (93/250) []d]	37.8 (34/90)	6.8 (5.4-8.2)[d]	6.8 (4.8-8.7)
			HR= 0.84 (0.70-1.02)	HR=0.73 (0.53-1.0) [c]				
ATTRACION-4	Nivo/Chemo	10.45 (8.44-14.75)	--	--	57.5 (208/362)	--	12.91 (9.89-16.56)	--
	Pl + SOX/CapOx	8.34 (6.97-9.40)	--	--	47.8 (173/362)	--	8.67 (7.2-11.37)	--
		HR = 0.68 (98.5% CI= 0.51-0.90)						
CheckMate649	Nivo.Chemo	7.7 (7.1–8.5)	7.5 (7.0–8.4)	7.7 (7.0–9.2)[b]	-	60(227/378)[b]	--	9.5 (8-11.4)[b]
	Folfox/CapOx	6.9 (6.6–7.1)	6.9 (6.1–7.0)	6 (5.6–6.9)		45 (176/391)	--	7 (5.7-7.9)
		HR=0.77(0.68-0.87)	HR= 0.74 (0.65-0.85)	HR= 0.68 (0.56-0.81)				
KEYNOTE-590	Pembro/ Chemo	6.3 (6.2-6.9)	--	7.5 mo (6.2-8.2) [c]	45 (168 /373)	--	8.3 (1.2-31)	--
	Pl+ Cis/5-Fu	5.8 (5.0-6.0)	--	5.5 mo (4.3-6.0)	29.3 (110 / 376)	--	6.0 (1.5-25)	--
		HR=0.65 (0.55-0.76)		HR= 0.51 (0.41-0.65)				
CheckMate-648	Nivo/Chemo	5.8 (5.6-7.0)	6.9 (5.7-78.3)	--	47	53[d]	8.2 (6.9-9.7)	8.4 (6.9-12.4)[d]
NCT03143153	Cis/5-Fu	5.6 (4.3-5.9)	4.4 (2.9-5.8)	--	27	20[d]	7.1 (5.7-8.2)	5.7 (4.4-8.7)[d]

		HR =0.81 (98.5% CI 0.64-1.04)	HR= 0.65 (98.5% CI 0.46-0.92)					
CheckMate-648	Nivo/Ipi	2.9 (2.7-4.2)	4.0 (2.4-4.9)	--	28	35[d]	11.1 (8.3-14.0)	11.8 (7.1-27.4)[d]
NCT03143153	Cis/5-Fu	5.6 (4.3-5.9)	4.4 (2.9-5.8)	--	27	20[d]	7.1 (5.7-8.2)	5.7 (4.4-8.7)[d]
		HR =1.26 (1.04-1.52)	HR=1.02 (98.5% CI 0.73-1.43)					
ESCORT-1st	Camre/Chemo	6.9 (5.8-7.4)	--	--	72.1 (214/298)	--	7.0 (6.1-8.9)	--
NCT03691090	Pac/Cis	5.6 (5.5-5.7)	--	--	62.1 (185/298)	--	4.6 (4.3-5.5)	--
		HR =0.56 (0.46-0.68)						

[a] Camre: camrelizumab; CapOx: capecitabine/oxaliplatin; Chemo: chemotherapy; CI: confidence interval; Cis: cisplatin; CPS = combined positive score; DOR = duration of response in months; E: esophageal; FOLFOX: folinic acid + fluorouracil + oxaliplatin; G = gastric; GEJ = gastroesophageal junction; IPI: ipilimumab; HR: hazard ratio; ms: months; Nivo: nivolumab; OS = overall survival; Pac: paclitaxe; PD-L1: programmed cell death ligand-1; Pembro: pembrolizumab; PFS = progression free survival; Pl: placebo; RR = response rate; SCC = squamous cell carcinoma; SOX = s1 (tegafur-gimeracil-oteracil potassium)/oxaliplatin; 5-Fu = 5-fluorouracil

[b] CPS expression ≥ 5%

[c] CPS expression ≥ 10%

[d] CPS expression ≥ 1%

Supplementary Table 3: Summary of Randomised trials in maintenance treatment (Overall Survival)[a]

Study	Line of Treatment	Tumor Site Histology	Arms	No. of patients	PD-L1 expression Any OS (95% CI), ms	PD-L1 expression CPS≥1 OS (95% CI), ms
JAVELIN-100	Maintenance	G/GEJ	Avelumab	249	10.4 (9.1–12.0)	16.2 (8.2–NR)
		AdenoCa	Folfox/Capox	250	10.9 (9.6–12.4)	17.7 (9.6–NR)
					HR= 0.91 (0.74-1.11)	HR: 1.13 (0.57- 2.23)
Bang et al.	Maintenance	G/GEJ	Ipilimumab	57	12.7 (10.5–18.9)	--
		AdenoCa	BSC[b]	57	12.1 (9.3–NE)	--
					HR= NA	
PLATFORM	Maintenance	E/G/ GEJ	Durvalumab	105	11.3 (8.7-14.0)	--
		AdenoCa	Surveillance	100	11.4 (7.9-13.6)	--
					HR=0.2: (0.66-1.27)	

[a] BSC: best supportive care; CapOx: capecitabine/oxaliplatin; Chemo: chemotherapy; CI: confidence interval; CPS = combined positive score; E: esophageal; FOLFOX: folinic acid + fluorouracil + oxaliplatin; G = gastric; GEJ = gastroesophageal junction; HR: hazard ratio; ms: months; NA = not applicable; NE: not estimated; NR: not reached; PD-L1: programmed cell death ligand-1;

[b] 79% had chemotherapy in the BSC arm.

Supplementary Table 4: Summary of Randomised trials in maintenance treatment (PFS, RR, DOR)[a]

Study	Arms	PD-L1 expression Any PFS (95% CI), ms	PD-L1 expression CPS≥1 PFS (95% CI), mos	irPFSms (95% CI)	RR (95% CI), %	DOR
JAVELIN-100	Avelumab	3.2 (2.8 to 4.1)	4.1 (1.6 to 16.0)	--	13.3 (9.3 -18.1)	NR (9.7 –NE)
	Folfox/Capox	4.4 (4.0 to 5.5)	9.7 (2.8 to 12.5)	--	14.4 (10.3 - 19.4)	5.9 (4.5 to 7.2)
		HR=1.04 (0.85-1.28)	HR= 1.04 (0.53 to 2.02)			
Bang et al	Ipilimumab	2.73 (1.45–2.96)	--	2.92 (1.61–5.16)	1.8 (0.91–1.00)	NA
	BSC	4.90 (3.45–6.08)	--	4.90 (3.45–6.54)	7.0 (0.83–0.98)	
		HR= 1.59 (80% CI= 1.20–2.10)		HR=1.44 (80% CI= 1.09–1.91		
PLATFORM	Durvalumab	4.7 (2.8-5.5)	--	--	0%	NA
	Surveillance	3.2 (2.8-5.2)	--	--	6%	
		HR= 0.79 (0.59-1.06)				

[a] CapOx: capecitabine/oxaliplatin; CI: confidence interval; CPS = combined positive score; DOR = Duration of Response; FOLFOX: folinic acid + fluorouracil + oxaliplatin; irPFS = Immunotherapy related progression-free survival; NA = not applicable; NE = not estimable; NR = Not Reached, PFS = progression free survival; RR = Response rate

Supplementary Table 5: Summary of Randomized trials in 2nd line treatment (Overall Survival)

Study	Line of Treatment	Tumor Site Histology	Arms	No. of patients	PD-L1 expression		
					Any<br>Median OS (95% CI), ms	CPS ≥ 1%<br>Median OS (95% CI), ms	CPS ≥ 10%<br>Median OS (95% CI), ms
ESCORT	2nd	Esophageal	Camrelizumab	228	8.3 (6.8-9.7)	9.2 (7-11.2)	NA
		SCC	Doc/Iri	220	6.2 (5.7-6.9)	6.3 (5.5-7.5)	NA
					HR=0.71 (0.57-0.87)	HR=0.58 (0.42-0.81)	HR=0.60 (0.32-1.08)
ATTRACTION-3	2nd	Esophageal	Nivolumab	210	10.9 (9.2-13.3)	NA	NA
		SCC	Pac/Doc	209	8.4 (7.2-9.9)		
					HR=0.77 (0.62-0.96)		
KEYNOTE-181	2nd	Esophageal	Pembrolizumab	314	7.1 (6.2-8.1)	NA	9.3 (6.6-12.6)
		AdenoCa/ SCC	Pac/Doc/Iri	314	7.1 (6.3-8.0)		6.7 (5.1-8.2)
					HR=0.89 (0.75-1.05)		HR=0.69 (0.52-0.93)
KEYNOTE-061	2nd	G/GEJ	Pembrolizumab	196	6.7 (5.4 – 8.9)	9.1 (6.2-10.7)	10.4 (5.9-18.3)
		AdenoCa	Paclitaxel	199	8.3 (7.7 – 8.8)	8.3 (7.6-9.0)	8.0 (5.1-9.9)
					HR=0.94 (0.79 – 1.12)	HR=0.81 (0.66-1.00)	HR=0.69 (0.46-1.05)
KEYNOTE-063	2nd	G/GEJ	Pembrolizumab	47	8.4 (4.0-9.5)	NA	NA
		AdenoCa	Paclitaxel	47	7.4 (5.4-11.3)		
					HR=NA		
RATIONALE-302	2nd	Esophageal	Tislelizumab	256	8.6 (7.5-10.4)	--	10.3 (8.5-16.1)
		SCC	Pac/Doc/Iri	256	6.3 (5.3-7.0)	--	6.8 (4.1-8.3)
					HR=0.70 (0.57-0.85)		HR=0.54 (0.36-0.79)

Kelly 2019	2nd& 3rd	G/GEJ	2L D+T	27	9.2 (5.4–12.6)	NA	NA
		AdenoCa	2L D	24	3.4 (1.7–4.4)		
			2L T	12	3.4 (1.7–4.4)		
			3L D+T	25	10.6 (4.8–14.8)		
			2L/3L D+TIFNγ	19	7.0 (2.4–7.5)		
					HR=NA		
CheckMate-032	1st, ≥ 2nd	Esophageal	NIVO3	59	6.2 (3.4 - 12.4)	NA	NA
		or G/GEJ	NIVO1 + IPI3	49	6.9 (3.7 - 11.5)		
		AdenoCa	NIVO3 + IPI1	52	4.8 (3.0 - 8.4)		
					HR= NA		

[a] AdenoCa: adenocarcinoma, D: Durvalumab; G: gastric; GEJ: gastroesophageal junction; CI: confidence interval; CPS: combined positive score; Doc: docetaxel; IFN: Interferon-γ; IPI3: ipilimumab 3 mg/kg; IPI1: ipilimumab 1 mg/kg; Iri: Irinotecan; HR: hazard ratio; ms: months; NA: not applicable; NIVO3: nivolumab 3mg/kg; NIVO1: nivolumab 1mg/kg; OS = overall survival, PD-L1: programmed cell death ligand-1; PFS = progression free survival, SCC: squamous cell carcinoma; T: Tremelimumab; 2L: second line; 3L: third line

Supplementary Table 6: Summary of Randomized trials in 2nd line treatment (PFS, RR, DOR)[a]

Study	PD-L1 expression			RR (n), %	Median DOR (95% CI), ms
	Any	CPS≥1	CPS≥ 10%		
	Median PFS (95% CI), ms	Median PFS (95% CI), ms	Median PFS (95% CI), mos		
ESCORT	1.9 (1.9-2.4)	NA	NA	20.2 (46/228)	7.4 (3.8-10.8)
	1.9 (1.9-2.1)			6.4 (14/220)	3.4 (0.9-not reached)
	HR=0.69 (0.56-0.86)				
ATTRACTION-3	1.7 (1.5-2.7)	NA	NA	19 (33/171)	6.9 (5.4-11.1)
	3.4 (3.0-4.2)			22 (34/158)	3.9 (2.8-4.2)
	HR=1.08 (0.87-1.34)				
KEYNOTE-181	2.1 (2.1-2.2)	NA	2.6 (2.1-4.1)	13.1 (41/314)	8.5 (NA)
	3.4 (2.8-3.9)		3.0 (2.1-3.7)	6.7 (21/314)	10.7 (NA)
	HR=1.11 (0.94-1.31)		HR= 0.73 (0.54-0.97)		
KEYNOTE-061	NA	1.5 (1.4-2.0)	2.7 (1.4-4.3)	16,3 (32/196)	19.1 (NA)
		4.1 (3.1-4.3)	4.0(2.7-4.4)	13.6 (27/199)	5.2 (NA)
		HR=1.25 (95% CI = 1.02-1.54)	HR= 0.79 (0.51-1.21)		
KEYNOTE-063	1.9(1.4-2.8)	NA	NA	12.8 (NA)	7.6 (NA)
	4.0 (2.7-6.2)			19.1 (NA)	12.4 (NA)
	HR= NA				
RATIONALE-302	NA	NA	NA	20.3 (52/256)	7.1 (4.1-11.3)
				9.8 (25/256)	4.0 (2.1-8.2)

Kelly et al 2019	1.8 (1.6–3.3)	NA	NA	2 (7,4%)	NA
	1.6 (1.0–1.8)			0 (0%)	NA
	1.7 (0.8–5.3)			1 (8,3%)	20.1 wks (NA- NA)
	1.8 (1.6–3.5)			1 (4%)	32.3 wks (NA- NA)
	1.8 (1.6–1.9)			3 (15,8%)	13.3 wks (8.1– NA)
	HR=NA				
CheckMate-032	1.4 (1.2 - 1.5)	NA	NA	4 (7%)	14.1 (2.8 - 14.1)
	1.4 (1.2 - 3.8)			10 (20%)	Not Reached
	1.6 (1.4 - 2.6)			2 (4%)	Not Reached
	HR=NA				

[a] CI: confidence interval; CPS: combined positive score; DOR = duration of response; HR: hazard ratio; ms: months; NA: not applicable; PFS = progression free survival, PD-L1: programmed cell death ligand-1; RR = response rate

Supplementary Figures

Supplementary Figure 1: Study Flow Chart for literature searches. ESMO = European Society of Medical Oncology; GI = gastrointestinal, ASCO = American Society of Clinical Oncology; *N Eng J Med = New England Journal of medicine; JCO = Journal of Clinical Oncology*; Oncol = Oncology

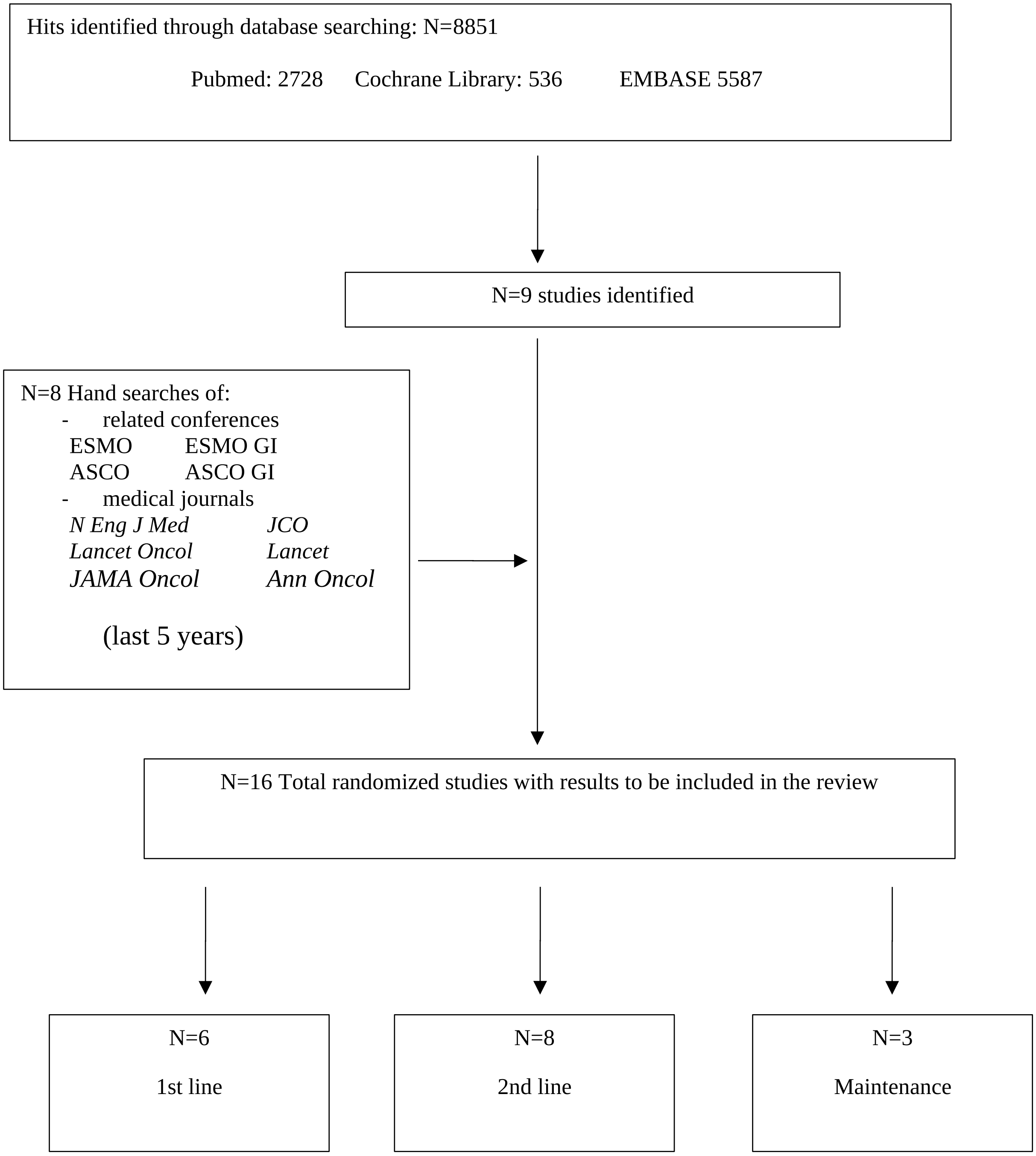

Supplementary Figure 2. Pooled Hazard Ratios for overall survival of 1st line studies evaluating immune checkpoint inhibitors in upper gastrointestinal malignancies. The error bars represent the 95% confidence intervals. A 2-sided p value < .05 was considered significant.

A)1st line, all studies included; B) 1st line, only patients with PD-L1 CPS ≥ 1% were included; C) 1st line, only patients with PD-L1 CPS ≥ 5 or 10% were included; D) 1st line, patients with any PD-L1 CPS were included (i.e. Keynote 062 reporting only patients with CPS >1 was excluded); E) 1st line, patients with adenocarcinomas were included; F) 1st line, patients with squamous cell carcinomas were included

The Inverse-Variance (IV) statistical method was applied for calculation of pooled Hazard Ratios (HRs). The Fixed Effects (FE) model was adopted to estimate the pooled ratios.

Abbreviations: PD-L1: Programmed death ligand 1; CPS: Combined positive score; IV: Inverse-Variance; HR: Hazard Ratio; CI: confidence intervals; FE: Fixed Effects

A

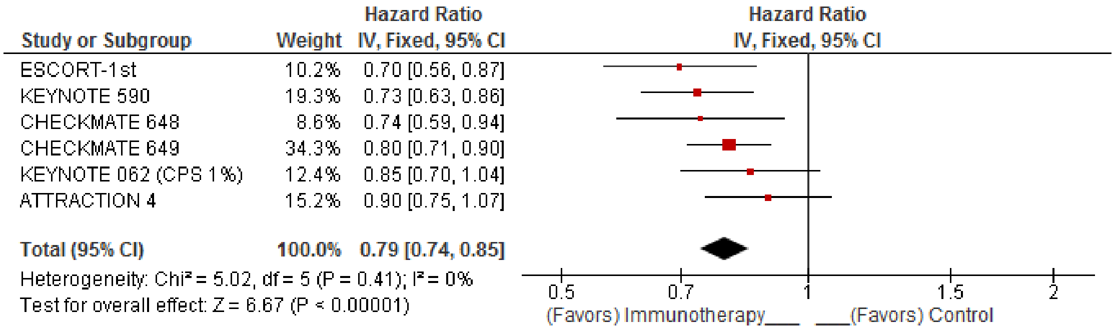

B

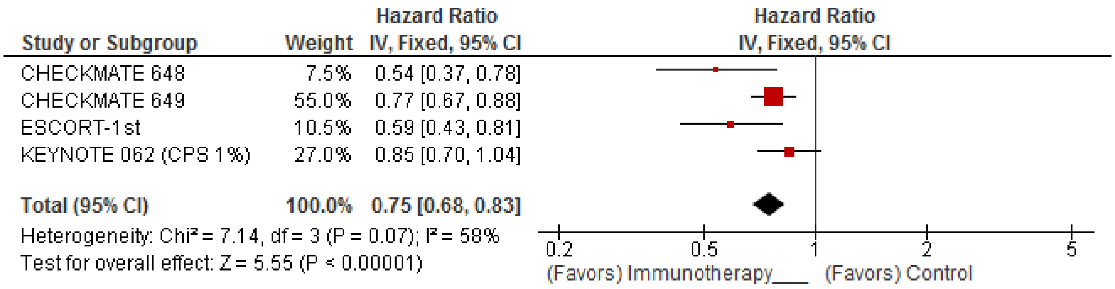

C

Study or Subgroup	Weight	Hazard Ratio IV, Fixed, 95% CI
CHECKMATE 649 (CPS 5%)	53.9%	0.71 [0.61, 0.83]
ESCORT-1st	8.6%	0.52 [0.35, 0.77]
KEYNOTE 062 (CPS 10%)	13.5%	0.85 [0.62, 1.17]
KEYNOTE 590 (CPS 10%)	24.0%	0.62 [0.49, 0.78]
**Total (95% CI)**	**100.0%**	**0.69 [0.61, 0.77]**

Heterogeneity: Chi² = 4.65, df = 3 (P = 0.20); I² = 36%
Test for overall effect: Z = 6.40 (P < 0.00001)

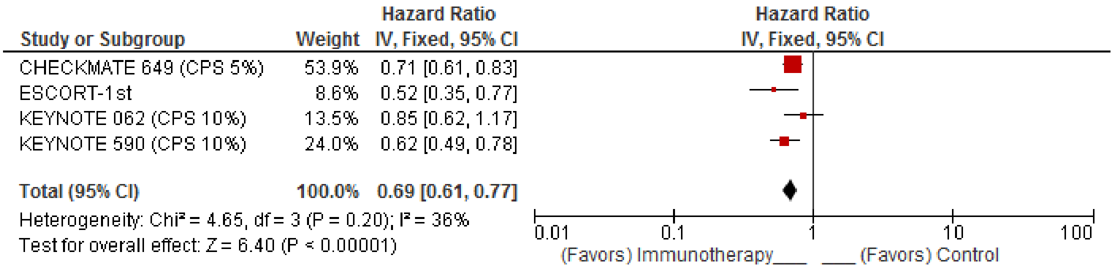

D

Study or Subgroup	Weight	Hazard Ratio IV, Fixed, 95% CI
ATTRACTION 4	17.4%	0.90 [0.75, 1.07]
CHECKMATE 648	9.8%	0.74 [0.59, 0.94]
CHECKMATE 649	39.1%	0.80 [0.71, 0.90]
ESCORT-1st	11.6%	0.70 [0.56, 0.87]
KEYNOTE 590	22.0%	0.73 [0.63, 0.86]
**Total (95% CI)**	**100.0%**	**0.78 [0.73, 0.84]**

Heterogeneity: Chi² = 4.39, df = 4 (P = 0.36); I² = 9%
Test for overall effect: Z = 6.52 (P < 0.00001)

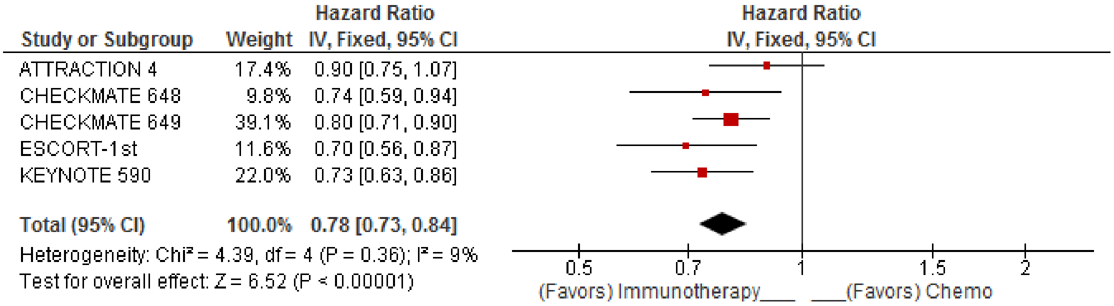

E

Study or Subgroup	Weight	Hazard Ratio IV, Fixed, 95% CI
ATTRACTION 4	22.8%	0.90 [0.75, 1.07]
CHECKMATE 649	51.4%	0.80 [0.71, 0.90]
KEYNOTE 062 (CPS 1%)	18.5%	0.85 [0.70, 1.04]
KEYNOTE 590 only adeno	7.2%	0.74 [0.54, 1.01]
**Total (95% CI)**	**100.0%**	**0.83 [0.76, 0.90]**

Heterogeneity: Chi² = 1.60, df = 3 (P = 0.66); I² = 0%
Test for overall effect: Z = 4.41 (P < 0.0001)

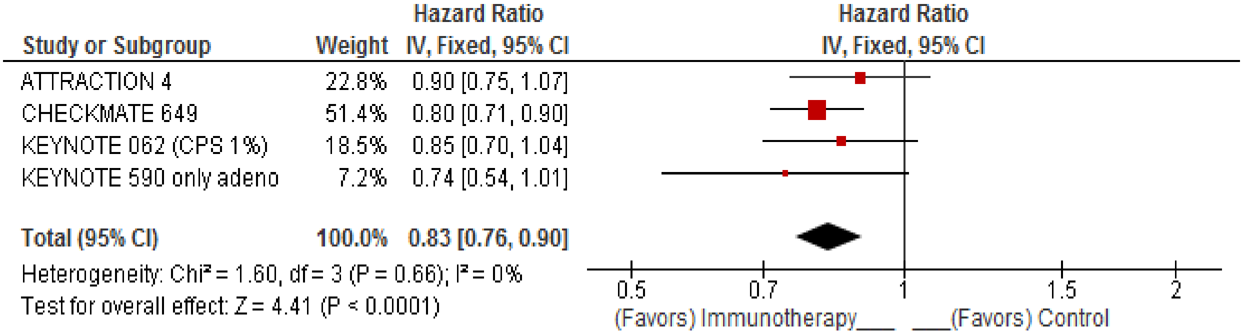

F

Study or Subgroup	Weight	Hazard Ratio IV, Fixed, 95% CI
CHECKMATE 648	44.8%	0.72 [0.60, 0.86]
ESCORT-1st	25.2%	0.74 [0.59, 0.94]
Keynote 590 (SCConly)	30.0%	0.70 [0.56, 0.87]
**Total (95% CI)**	**100.0%**	**0.72 [0.64, 0.81]**

Heterogeneity: Chi² = 0.14, df = 2 (P = 0.93); I² = 0%
Test for overall effect: Z = 5.50 (P < 0.00001)

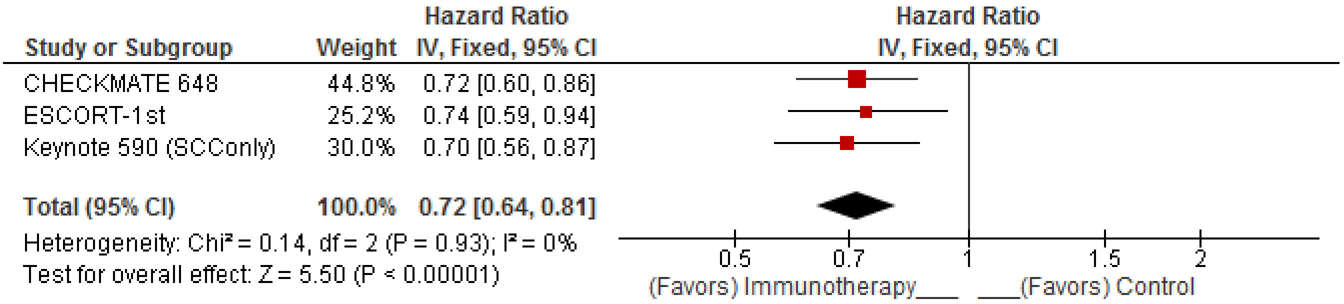

Supplementary Figure 3. Pooled Hazard Ratios for Progression Free Survival of 1st line studies evaluating immune checkpoint inhibitors in upper gastrointestinal malignancies. The error bars represent the 95% confidence intervals. A 2-sided p value < .05 was considered significant.

A)1st line, all studies included; B) 1st line, patients with PD-L1 CPS ≥ 1% were included; C) 1st line, patients with PD-L1 CPS ≥ 5 or 10% were included; D) 1st line, patients with any PD-L1 CPS were included (i.e. Keynote 062 reporting only patients with CPS >1 was excluded); E) 1st line, patients with adenocarcinomas were included; F) 1st line, patients with squamous cell carcinomas were included.

The Inverse-Variance (IV) statistical method was applied for calculation of pooled Hazard Ratios (HRs). In case of statistically significant heterogeneity (Q test P<0.1) the Random Effects (RE) model was reported; otherwise the Fixed Effects (FE) model was adopted to estimate the pooled ratios.

Abbreviations: PD-L1: Programmed death ligand 1; CPS: Combined positive score; IV: Inverse-Variance; HR: Hazard Ratio; CI: confidence intervals; FE: Fixed Effects; RE: Random Effects

A.

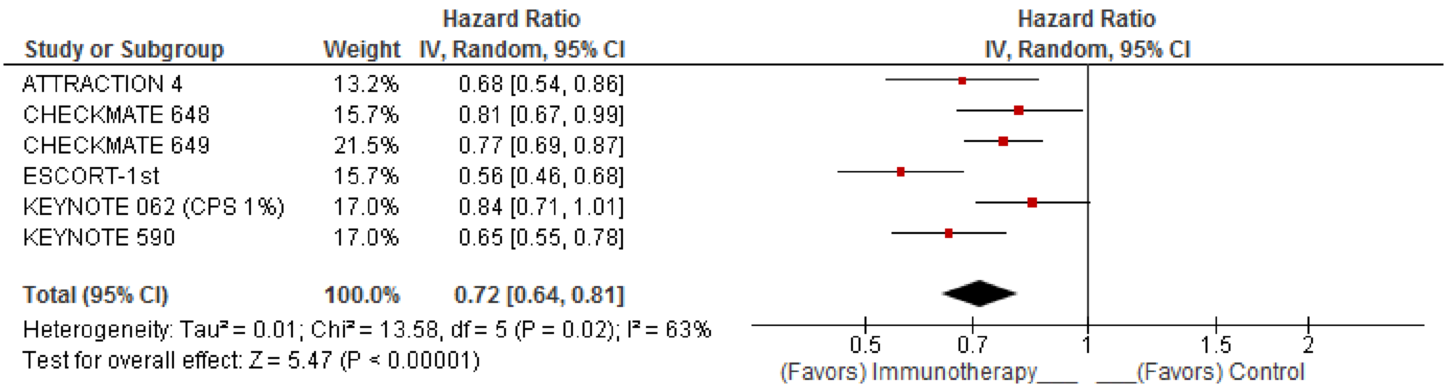

B.

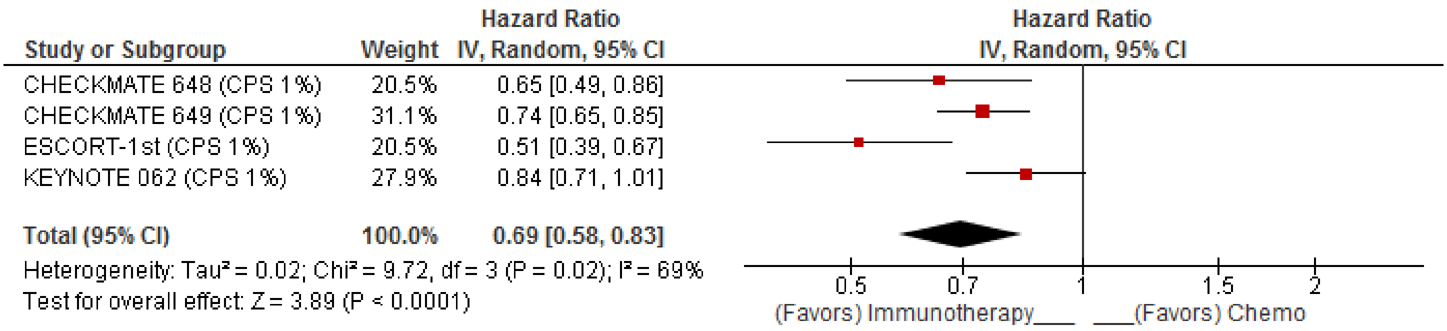

C.

Study or Subgroup	Weight	Hazard Ratio IV, Fixed, 95% CI
CHECKMATE 649 (CPS 5%)	50.6%	0.68 [0.58, 0.79]
ESCORT-1st (CPS 10%)	10.0%	0.51 [0.36, 0.73]
KEYNOTE 062 (CPS 10%)	12.6%	0.73 [0.54, 1.00]
KEYNOTE 590 (CPS 10%)	26.8%	0.51 [0.41, 0.63]
**Total (95% CI)**	**100.0%**	**0.62 [0.55, 0.69]**

Heterogeneity: Chi² = 6.49, df = 3 (P = 0.09); I² = 54%
Test for overall effect: Z = 8.48 (P < 0.00001)

Hazard Ratio IV, Fixed, 95% CI

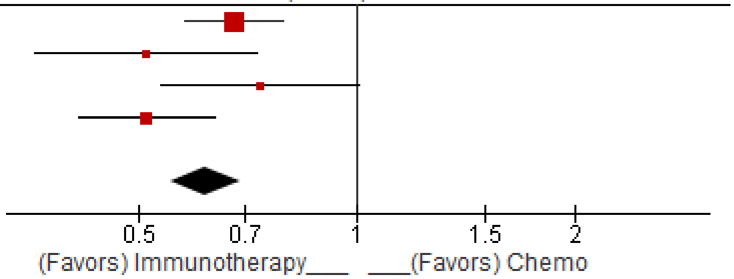

D.

Study or Subgroup	Weight	Hazard Ratio IV, Random, 95% CI
ATTRACTION 4	15.8%	0.68 [0.54, 0.86]
CHECKMATE 648	18.8%	0.81 [0.67, 0.99]
CHECKMATE 649	26.1%	0.77 [0.69, 0.87]
ESCORT-1st	18.8%	0.56 [0.46, 0.68]
KEYNOTE 590	20.5%	0.65 [0.55, 0.78]
**Total (95% CI)**	**100.0%**	**0.69 [0.61, 0.79]**

Heterogeneity: Tau² = 0.01; Chi² = 10.38, df = 4 (P = 0.03); I² = 61%
Test for overall effect: Z = 5.58 (P < 0.00001)

Hazard Ratio IV, Random, 95% CI

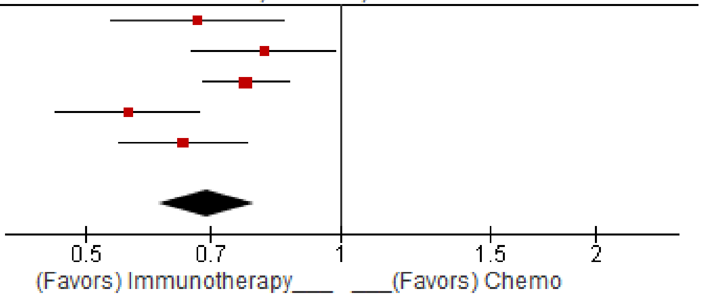

E.

Study or Subgroup	Weight	Hazard Ratio IV, Fixed, 95% CI
ATTRACTION 4	13.6%	0.68 [0.54, 0.86]
CHECKMATE 649	54.5%	0.77 [0.69, 0.87]
KEYNOTE 062 (CPS 1%)	24.2%	0.84 [0.71, 1.01]
KEYNOTE 590 only adeno	7.7%	0.63 [0.46, 0.86]
**Total (95% CI)**	**100.0%**	**0.76 [0.70, 0.83]**

Heterogeneity: Chi² = 3.67, df = 3 (P = 0.30); I² = 18%
Test for overall effect: Z = 6.12 (P < 0.00001)

Hazard Ratio IV, Fixed, 95% CI

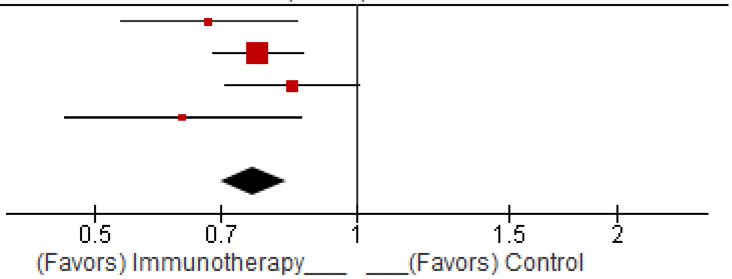

F.

Study or Subgroup	Weight	Hazard Ratio IV, Random, 95% CI
CHECKMATE 648	29.7%	0.81 [0.67, 0.99]
ESCORT-1st	29.7%	0.56 [0.46, 0.68]
Keynote 590 (SCConly)	40.6%	0.72 [0.65, 0.79]
**Total (95% CI)**	**100.0%**	**0.69 [0.58, 0.83]**

Heterogeneity: Tau² = 0.02; Chi² = 7.41, df = 2 (P = 0.02); I² = 73%
Test for overall effect: Z = 4.04 (P < 0.0001)

Hazard Ratio IV, Random, 95% CI

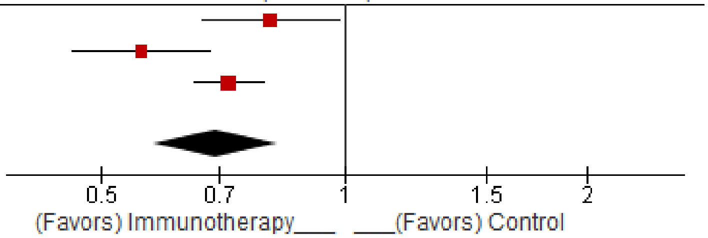

Supplementary Figure 4. Pooled Odds Ratio for Objective Response Rate of 1st line studies evaluating immune checkpoint inhibitors in upper gastrointestinal malignancies. The error bars represent the 95% confidence intervals. A 2-sided p value < .05 was considered significant.

A) all studies included; B) patients with any PD-L1 CPS were included (i.e. Checkmate 649 and Keynote 062 were excluded); C) patients with PD-L1 CPS ≥ 5% or 10% were included.

The Mantel-Haenszel (MH) statistical method was applied for calculation of pooled odds ratios. No heterogeneity was detected, so the Fixed Effects (FE) model was adopted to estimate the pooled ratios.

Abbreviations: PD-L1: Programmed death ligand 1; CPS: Combined positive score; MH: Mantel-Haenszel; OR: Odds Ratio; CI: confidence intervals; FE: Fixed Effects.

A.

	IO&CHEMO		CHEMO			Odds Ratio
Study or Subgroup	Events	Total	Events	Total	Weight	M-H, Fixed, 95% CI
ATTRACTION 4	208	362	173	362	29.3%	1.48 [1.10, 1.98]
CHECKMATE 649 (CPS 5%)	227	378	176	391	27.5%	1.84 [1.38, 2.45]
KEYNOTE 062 (CPS 1%)	125	257	93	250	19.3%	1.60 [1.12, 2.28]
KEYNOTE 590	168	373	110	376	24.0%	1.98 [1.47, 2.68]
**Total (95% CI)**		**1370**		**1379**	**100.0%**	**1.72 [1.48, 2.00]**
Total events	728		552			

Heterogeneity: Chi² = 2.26, df = 3 (P = 0.52); I² = 0%

Test for overall effect: Z = 6.96 (P < 0.00001)

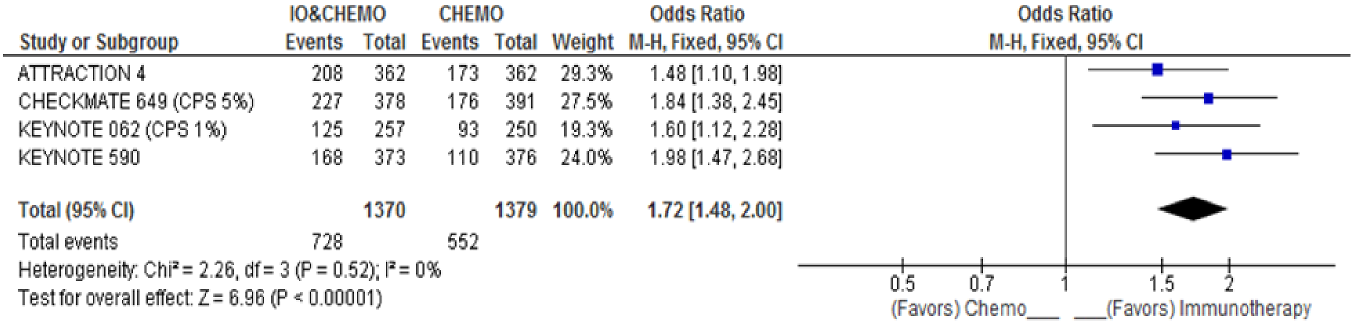

B.

	IO&CHEMO		CHEMO			Odds Ratio
Study or Subgroup	Events	Total	Events	Total	Weight	M-H, Fixed, 95% CI
ATTRACTION 4	208	362	173	362	55.0%	1.48 [1.10, 1.98]
KEYNOTE 590	168	373	110	376	45.0%	1.98 [1.47, 2.68]
**Total (95% CI)**		**735**		**738**	**100.0%**	**1.70 [1.38, 2.10]**
Total events	376		283			

Heterogeneity: Chi² = 1.89, df = 1 (P = 0.17); I² = 47%

Test for overall effect: Z = 4.97 (P < 0.00001)

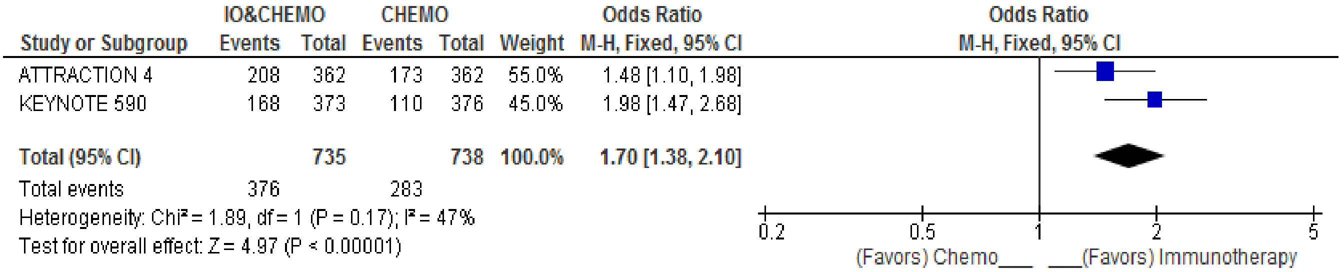

C.

	IO&CHEMO		CHEMO			Odds Ratio
Study or Subgroup	Events	Total	Events	Total	Weight	M-H, Fixed, 95% CI
CHECKMATE 649 (CPS 5%)	227	378	176	391	80.3%	1.84 [1.38, 2.45]
KEYNOTE 062 (CPS 10%)	52	99	34	90	19.7%	1.82 [1.02, 3.26]
**Total (95% CI)**		**477**		**481**	**100.0%**	**1.83 [1.42, 2.37]**
Total events	279		210			

Heterogeneity: Chi² = 0.00, df = 1 (P = 0.98); I² = 0%

Test for overall effect: Z = 4.63 (P < 0.00001)

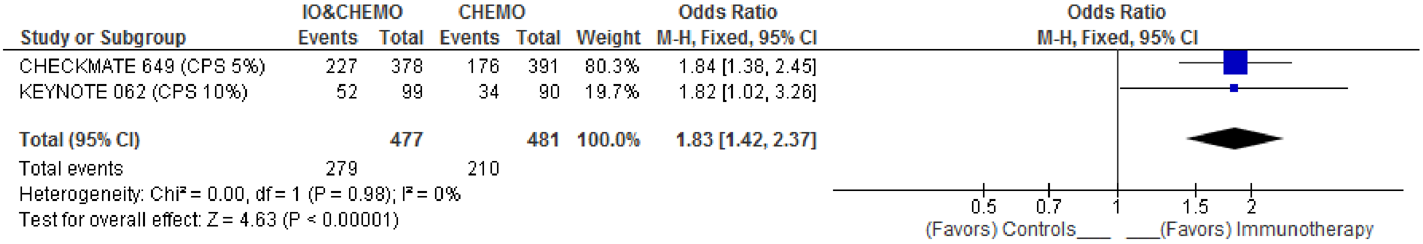

Supplementary Figure 5. Pooled Hazard Ratio for Overall Survival of studies evaluating maintenance with immune checkpoint inhibitors in upper gastrointestinal malignancies. The error bars represent the 95% confidence intervals. A 2-sided p value < .05 was considered significant.

The Inverse-Variance (IV) statistical method was applied for calculation of pooled Hazard Ratio (HR). No heterogeneity was detected, so the Fixed Effects (FE) model was adopted to estimate the pooled ratio.

Abbreviations: IV: Inverse-Variance; HR: Hazard Ratio; CI: confidence intervals; FE: Fixed Effects

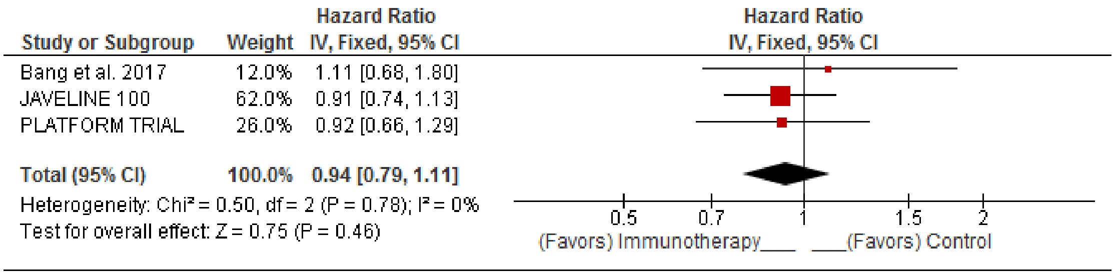

Supplementary Figure 6. Pooled Hazard Ratio for Progression Free Survival of studies evaluating maintenance with immune checkpoint inhibitors in upper gastrointestinal malignancies. The error bars represent the 95% confidence intervals. A 2-sided p value < .05 was considered significant.

The Inverse-Variance (IV) statistical method was applied for calculation of pooled Hazard Ratio (HR). Heterogeneity was detected, so the Random Effects (RE) model was adopted to estimate the pooled ratio. The 95% confidence intervals (CIs) were used for the analysis.

Abbreviations: IV: Inverse-Variance; HR: Hazard Ratio; CI: confidence intervals; RE: Random Effects

Study or Subgroup	Weight	Hazard Ratio IV, Random, 95% CI
Bang et al. 2017	18.4%	1.58 [0.90, 2.80]
JAVELINE 100	45.2%	1.04 [0.86, 1.27]
PLATFORM TRIAL	36.3%	0.79 [0.59, 1.06]
**Total (95% CI)**	**100.0%**	**1.02 [0.75, 1.37]**

Heterogeneity: $Tau^2 = 0.04$; $Chi^2 = 5.23$, df = 2 (P = 0.07); $I^2 = 62\%$
Test for overall effect: Z = 0.10 (P = 0.92)

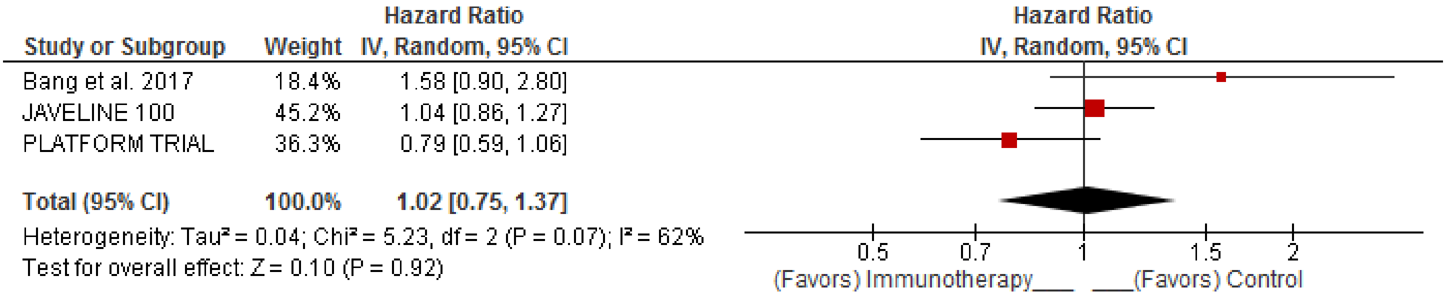

Supplementary Figure 7. Pooled Odds Ratio for Objective Response Rate of studies evaluating maintenance with immune checkpoint inhibitors in upper gastrointestinal malignancies. The error bars represent the 95% confidence intervals. A 2-sided p value < .05 was considered significant.

The Mantel-Haenszel (MH) statistical method was applied for calculation of pooled OR. No heterogeneity was detected, so the Fixed Effects (FE) model was adopted to estimate the pooled ratio.

Abbreviations: MH: Mantel-Haenszel; OR: Odds Ratio; CI: confidence intervals; FE: Fixed Effects.

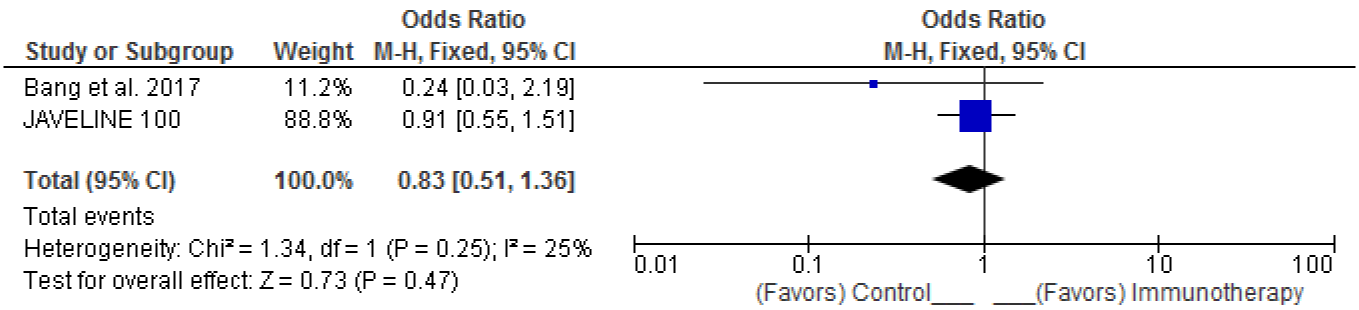

Supplementary Figure 8. Pooled Hazard Ratios for overall survival of 2nd line studies evaluating immune checkpoint inhibitors in upper gastrointestinal malignancies. The error bars represent the 95% confidence intervals. A 2-sided p value < .05 was considered significant.

A) 2nd line, all studies included; B) 2nd line, patients with PD-L1 CPS ≥ 1% were included; C) 2nd line, patients with PD-L1 CPS ≥ 10% were included; D) 2nd line, patients with squamous cell carcinomas were included; E) 2nd line, patients with adenocarcinomas were included; F) 2nd line, patients with adenocarcinomas and PD-L1 CPS ≥ 10% were included; G) 2nd line, patients with squamous cell carcinomas and PD-L1 CPS ≥ 1% were included; H) 2nd line, patients with squamous cell carcinomas and PD-L1 CPS ≥ 10% were included; I) 2nd line, patients with PD-L1 CPS < 1% were included; J) 2nd line, patients with squamous cell carcinomas and PD-L1 CPS < 5% were included; K) 2nd line, patients with squamous cell carcinomas and PD-L1 CPS < 10% were included; L) 2nd line, patients with PD-L1 CPS < 10%, any histology were included; M) 2nd line, patients with squamous cell carcinomas and PD-L1 CPS < 1% were included.

The Inverse-Variance (IV) statistical method was applied for calculation of pooled Hazard Ratios (HRs). In case of statistically significant heterogeneity (Q test P<0.1) the Random Effects (RE) model was reported; otherwise, the Fixed Effects (FE) model was adopted to estimate the pooled ratios.

Abbreviations: PD-L1: Programmed death ligand 1; CPS: Combined positive score; IV: Inverse-Variance; HR: Hazard Ratio; CI: confidence intervals; FE: Fixed Effects; RE: Random Effects

A.

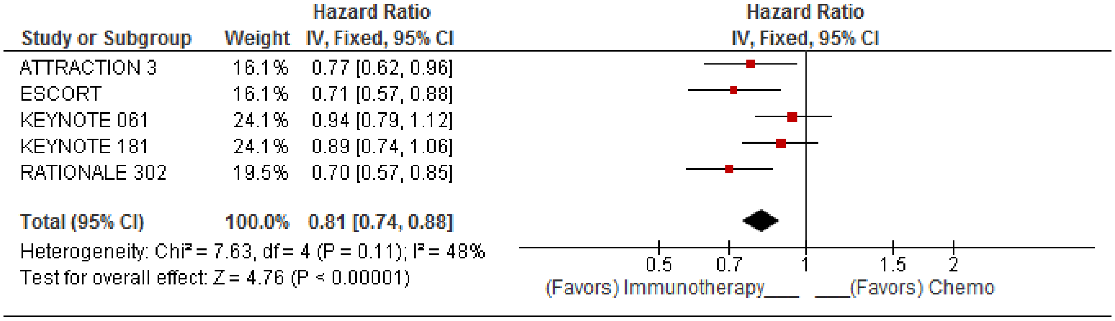

B.

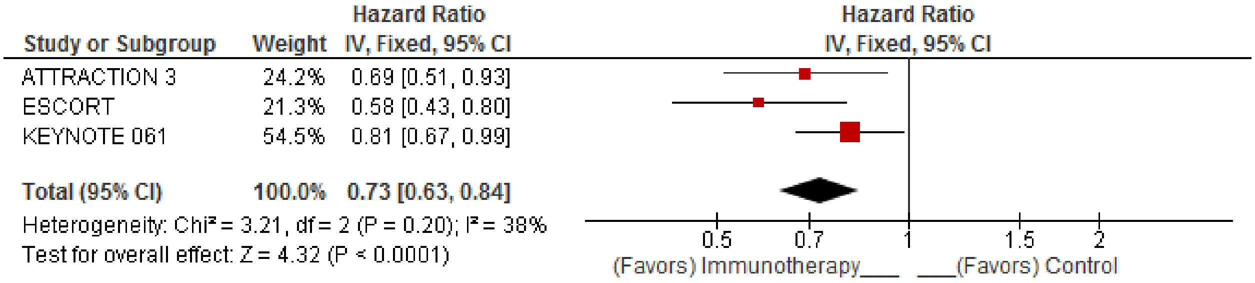

C.

Study or Subgroup	Weight	Hazard Ratio IV, Fixed, 95% CI
ATTRACTION 3	17.6%	0.69 [0.46, 1.04]
ESCORT	7.6%	0.60 [0.32, 1.12]
KEYNOTE 061	17.6%	0.69 [0.46, 1.04]
KEYNOTE 181	39.6%	0.69 [0.52, 0.91]
RATIONALE 302	17.6%	0.54 [0.36, 0.81]
**Total (95% CI)**	**100.0%**	**0.65 [0.55, 0.78]**

Heterogeneity: Chi² = 1.22, df = 4 (P = 0.87); I² = 0%
Test for overall effect: Z = 4.82 (P < 0.00001)

Hazard Ratio
IV, Fixed, 95% CI

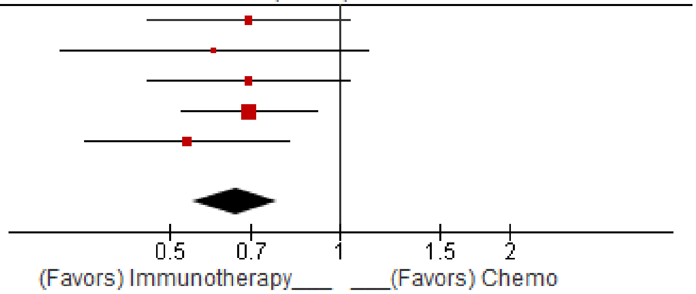

D.

Study or Subgroup	Weight	Hazard Ratio IV, Fixed, 95% CI
ATTRACTION 3	22.6%	0.77 [0.62, 0.96]
ESCORT	22.6%	0.71 [0.57, 0.88]
KEYNOTE 181 only SCC	27.4%	0.77 [0.63, 0.94]
RATIONALE 302	27.4%	0.70 [0.57, 0.85]
**Total (95% CI)**	**100.0%**	**0.74 [0.66, 0.82]**

Heterogeneity: Chi² = 0.77, df = 3 (P = 0.86); I² = 0%
Test for overall effect: Z = 5.84 (P < 0.00001)

Hazard Ratio
IV, Fixed, 95% CI

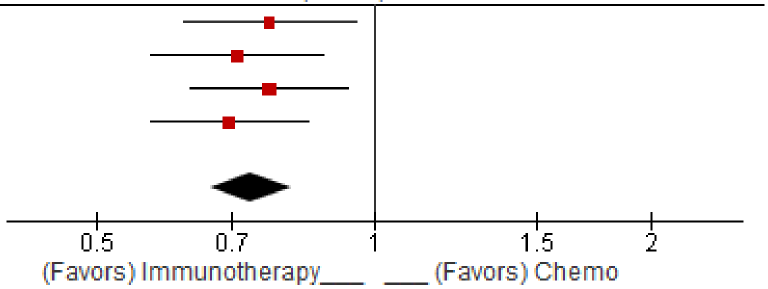

E.

Study or Subgroup	Weight	Hazard Ratio IV, Fixed, 95% CI
KEYNOTE 061	70.8%	0.94 [0.79, 1.12]
KEYNOTE 181 only adeno	29.2%	1.12 [0.85, 1.47]
**Total (95% CI)**	**100.0%**	**0.99 [0.85, 1.15]**

Heterogeneity: Chi² = 1.04, df = 1 (P = 0.31); I² = 4%
Test for overall effect: Z = 0.14 (P = 0.89)

Hazard Ratio
IV, Fixed, 95% CI

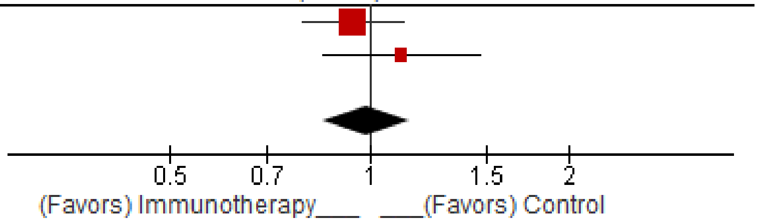

F.

Study or Subgroup	Weight	Hazard Ratio IV, Fixed, 95% CI
KEYNOTE 061	67.1%	0.69 [0.46, 1.04]
KEYNOTE 181	32.9%	0.93 [0.52, 1.68]
**Total (95% CI)**	**100.0%**	**0.76 [0.54, 1.07]**

Heterogeneity: Chi² = 0.67, df = 1 (P = 0.41); I² = 0%
Test for overall effect: Z = 1.58 (P = 0.11)

Hazard Ratio
IV, Fixed, 95% CI

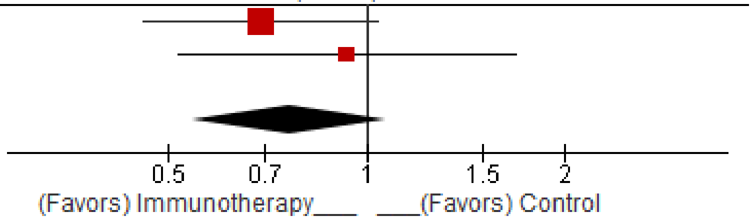

G.

Study or Subgroup	Weight	Hazard Ratio IV, Fixed, 95% CI
ATTRACTION 3	53.2%	0.69 [0.51, 0.93]
ESCORT	46.8%	0.58 [0.43, 0.80]
**Total (95% CI)**	**100.0%**	**0.64 [0.51, 0.79]**

Heterogeneity: $Chi^2 = 0.60, df = 1 (P = 0.44); I^2 = 0\%$
Test for overall effect: $Z = 4.11 (P < 0.0001)$

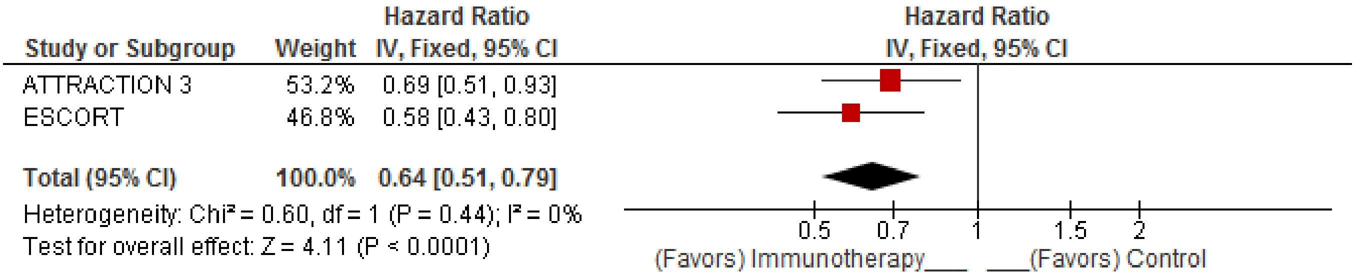

H.

Study or Subgroup	Weight	Hazard Ratio IV, Fixed, 95% CI
ATTRACTION 3	25.3%	0.69 [0.46, 1.04]
ESCORT	10.9%	0.60 [0.32, 1.12]
KEYNOTE 181	38.6%	0.64 [0.46, 0.89]
RATIONALE 302	25.3%	0.54 [0.36, 0.81]
**Total (95% CI)**	**100.0%**	**0.62 [0.50, 0.76]**

Heterogeneity: $Chi^2 = 0.76, df = 3 (P = 0.86); I^2 = 0\%$
Test for overall effect: $Z = 4.54 (P < 0.00001)$

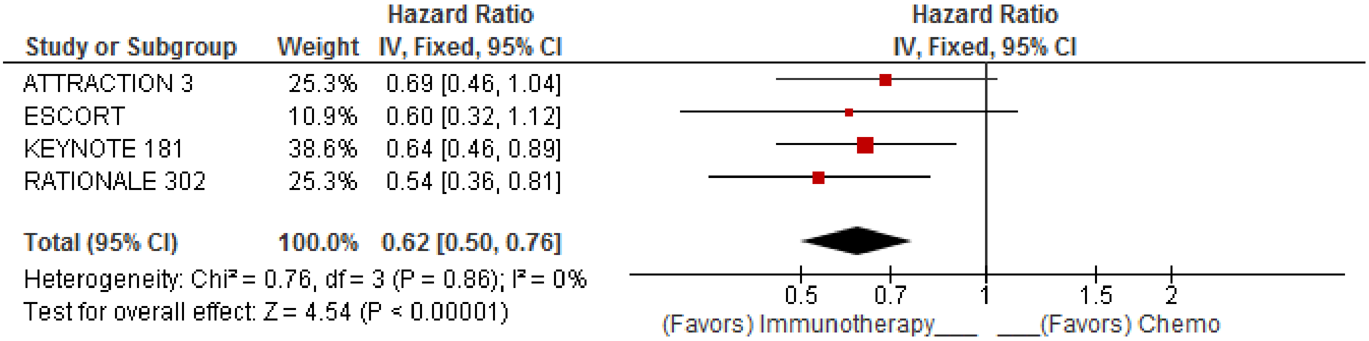

I.

Study or Subgroup	Weight	Hazard Ratio IV, Fixed, 95% CI
ATTRACTION 3	31.8%	0.84 [0.63, 1.13]
ESCORT	36.5%	0.82 [0.62, 1.08]
KEYNOTE 061	31.8%	1.20 [0.89, 1.61]
**Total (95% CI)**	**100.0%**	**0.93 [0.79, 1.10]**

Heterogeneity: $Chi^2 = 4.08, df = 2 (P = 0.13); I^2 = 51\%$
Test for overall effect: $Z = 0.83 (P = 0.41)$

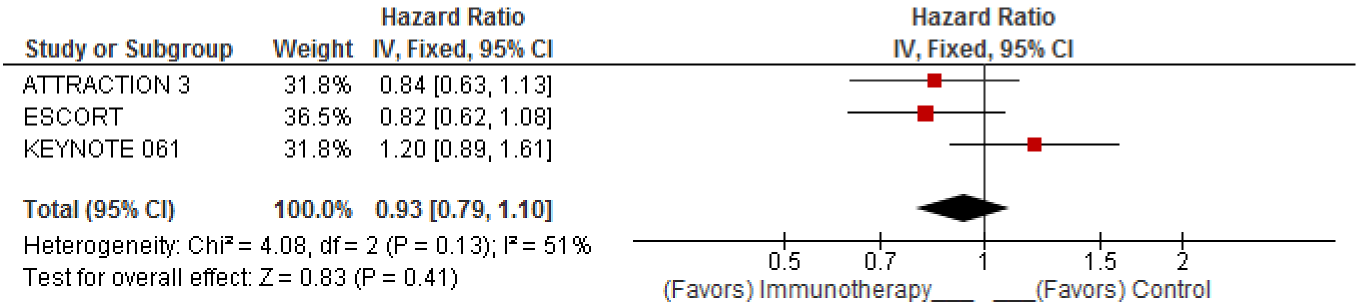

J.

Study or Subgroup	Weight	Hazard Ratio IV, Fixed, 95% CI
ATTRACTION 3	46.0%	0.78 [0.60, 1.00]
ESCORT	54.0%	0.74 [0.59, 0.94]
**Total (95% CI)**	**100.0%**	**0.76 [0.64, 0.90]**

Heterogeneity: $Chi^2 = 0.08, df = 1 (P = 0.78); I^2 = 0\%$
Test for overall effect: $Z = 3.14 (P = 0.002)$

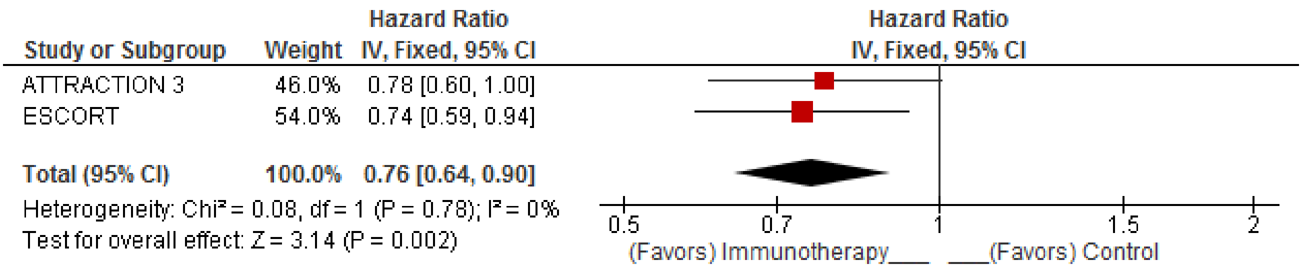

K.

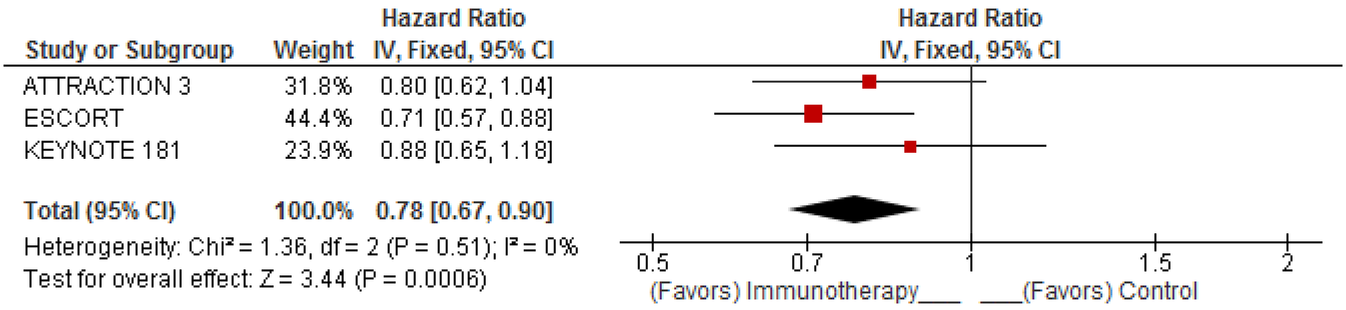
Hazard Ratio
Study or Subgroup
Weight
IV, Fixed, 95% CI
Hazard Ratio
IV, Fixed, 95% CI
ATTRACTION 3 31.8% 0.80 [0.62, 1.04]
ESCORT 44.4% 0.71 [0.57, 0.88]
KEYNOTE 181 23.9% 0.88 [0.65, 1.18]
Total (95% CI) 100.0% 0.78 [0.67, 0.90]
Heterogeneity: Chi² = 1.36, df = 2 (P = 0.51); I² = 0%
Test for overall effect: Z = 3.44 (P = 0.0006)
0.5
0.7
1
1.5
2
(Favors) Immunotherapy
(Favors) Control

L.

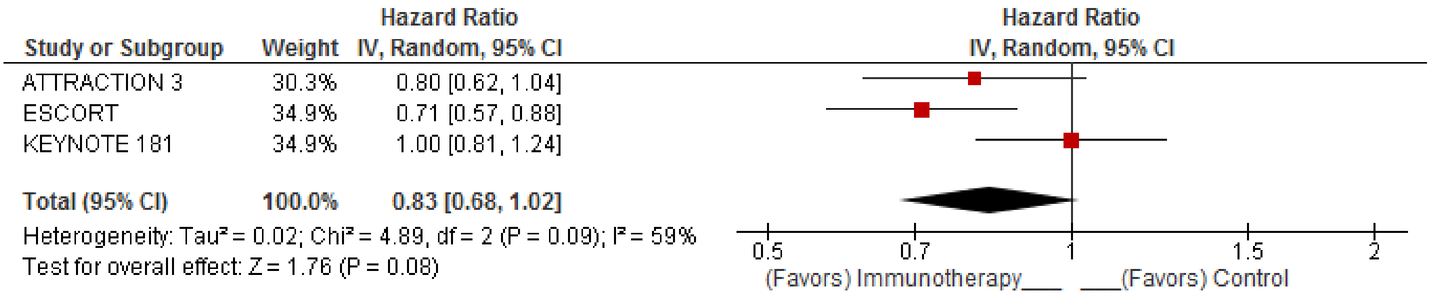
Hazard Ratio
Study or Subgroup
Weight
IV, Random, 95% CI
Hazard Ratio
IV, Random, 95% CI
ATTRACTION 3 30.3% 0.80 [0.62, 1.04]
ESCORT 34.9% 0.71 [0.57, 0.88]
KEYNOTE 181 34.9% 1.00 [0.81, 1.24]
Total (95% CI) 100.0% 0.83 [0.68, 1.02]
Heterogeneity: Tau² = 0.02; Chi² = 4.89, df = 2 (P = 0.09); I² = 59%
Test for overall effect: Z = 1.76 (P = 0.08)
0.5
0.7
1
1.5
2
(Favors) Immunotherapy
(Favors) Control

M.

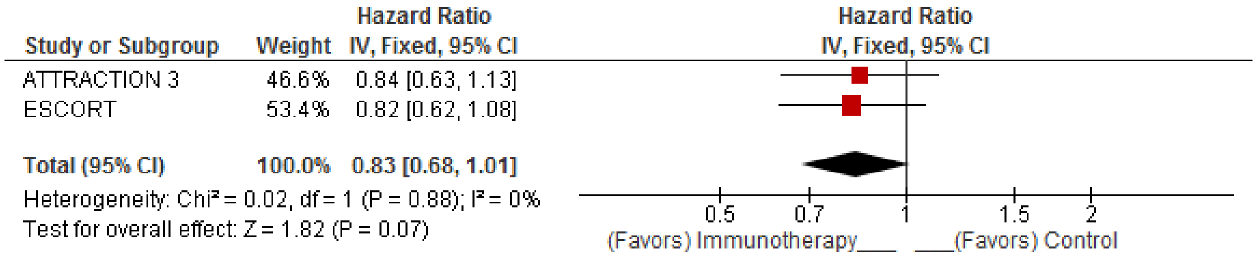
Hazard Ratio
Study or Subgroup
Weight
IV, Fixed, 95% CI
Hazard Ratio
IV, Fixed, 95% CI
ATTRACTION 3 46.6% 0.84 [0.63, 1.13]
ESCORT 53.4% 0.82 [0.62, 1.08]
Total (95% CI) 100.0% 0.83 [0.68, 1.01]
Heterogeneity: Chi² = 0.02, df = 1 (P = 0.88); I² = 0%
Test for overall effect: Z = 1.82 (P = 0.07)
0.5
0.7
1
1.5
2
(Favors) Immunotherapy
(Favors) Control

Supplementary Figure 9. Pooled Hazard Ratio for Progression Free Survival of 2nd line studies evaluating immune checkpoint inhibitors in upper gastrointestinal malignancies. The error bars represent the 95% confidence intervals. A 2-sided p value < .05 was considered significant.

A) 2nd line, all studies were included; B) 2nd line, patients with PD-L1 CPS > 1% were included; C) 2nd line, patients with PD-L1 CPS ≥ 10% were included; D) 2nd line, patients with squamous cell carcinomas were included; E) 2nd line, patients with PD-L1 CPS < 1% were included; F) 2nd line, only international studies were included

The Inverse-Variance (IV) statistical method was applied for calculation of pooled Hazard Ratios (HRs). In case of statistically significant heterogeneity (Q test P<0.1) the Random Effects (RE) model was reported; otherwise, the Fixed Effects (FE) model was adopted to estimate the pooled ratios.

Abbreviations: PD-L1: Programmed death ligand 1; CPS: Combined positive score; IV: Inverse-Variance; HR: Hazard Ratio; CI: confidence intervals; FE: Fixed Effects; RE: Random Effects

A.

Study or Subgroup	Weight	Hazard Ratio IV, Random, 95% CI
ATTRACTION 3	24.3%	1.08 [0.87, 1.34]
ESCORT	24.3%	0.69 [0.56, 0.86]
KEYNOTE 061	25.4%	1.49 [1.25, 1.78]
KEYNOTE 181	25.9%	1.11 [0.94, 1.29]
**Total (95% CI)**	**100.0%**	**1.06 [0.79, 1.42]**

Heterogeneity: Tau² = 0.08; Chi² = 29.37, df = 3 (P < 0.00001); I² = 90%
Test for overall effect: Z = 0.38 (P = 0.70)

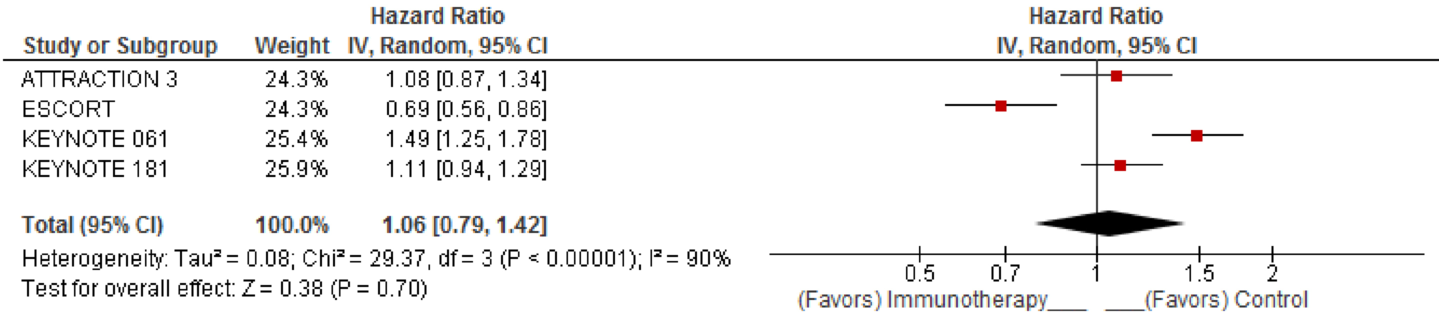

B.

Study or Subgroup	Weight	Hazard Ratio IV, Random, 95% CI
ESCORT	48.2%	0.60 [0.43, 0.84]
KEYNOTE 061	51.8%	1.25 [1.02, 1.52]
**Total (95% CI)**	**100.0%**	**0.88 [0.43, 1.79]**

Heterogeneity: Tau² = 0.25; Chi² = 13.70, df = 1 (P = 0.0002); I² = 93%
Test for overall effect: Z = 0.36 (P = 0.72)

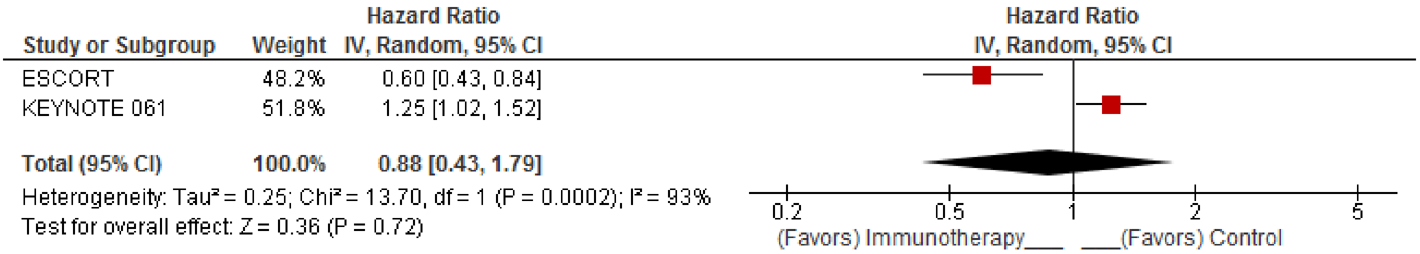

C.

Study or Subgroup	Weight	Hazard Ratio IV, Fixed, 95% CI
ESCORT	11.7%	0.47 [0.24, 0.91]
KEYNOTE 061	28.0%	0.79 [0.51, 1.21]
KEYNOTE 181	60.3%	0.73 [0.55, 0.98]
**Total (95% CI)**	**100.0%**	**0.71 [0.56, 0.89]**

Heterogeneity: Chi² = 1.77, df = 2 (P = 0.41); I² = 0%
Test for overall effect: Z = 2.95 (P = 0.003)

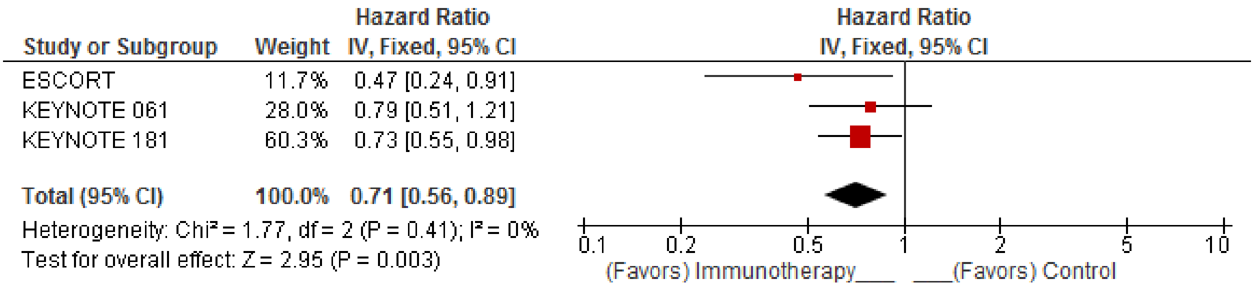

D.

Study or Subgroup	Weight	Hazard Ratio IV, Random, 95% CI
ATTRACTION 3	32.9%	1.08 [0.87, 1.34]
ESCORT	32.9%	0.69 [0.56, 0.86]
KEYNOTE 181 only SCC	34.3%	0.92 [0.76, 1.12]
**Total (95% CI)**	**100.0%**	**0.88 [0.69, 1.14]**

Heterogeneity: Tau² = 0.04; Chi² = 8.63, df = 2 (P = 0.01); I² = 77%
Test for overall effect: Z = 0.96 (P = 0.34)

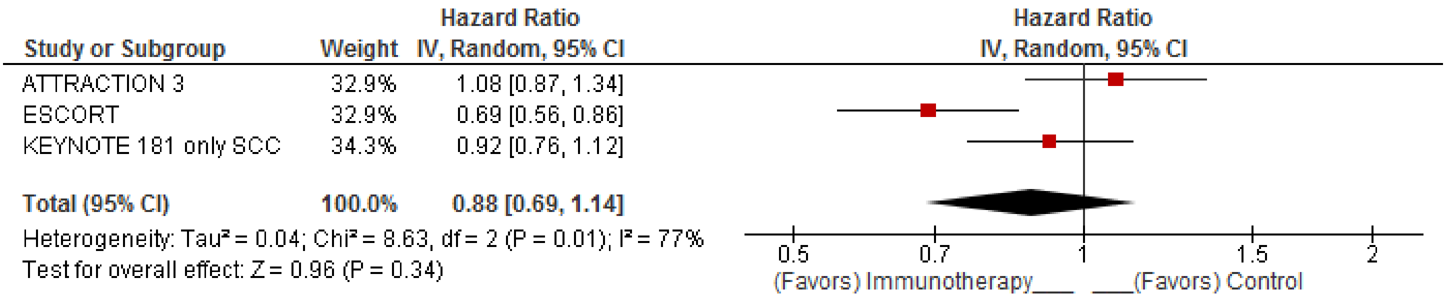

E.

Study or Subgroup	Weight	Hazard Ratio IV, Random, 95% CI
ESCORT	50.2%	0.79 [0.59, 1.06]
KEYNOTE 061	49.8%	2.05 [1.50, 2.81]
**Total (95% CI)**	**100.0%**	**1.27 [0.50, 3.25]**

Heterogeneity: Tau² = 0.44; Chi² = 19.16, df = 1 (P < 0.0001); I² = 95%
Test for overall effect: Z = 0.50 (P = 0.62)

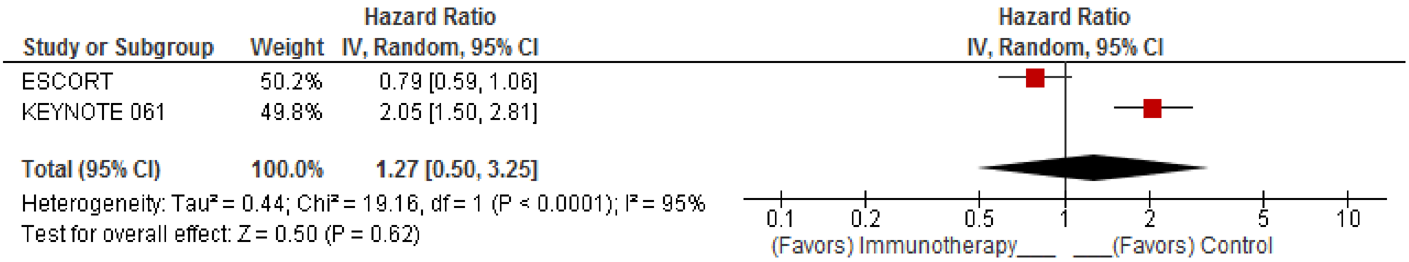

F.

Study or Subgroup	Weight	Hazard Ratio IV, Random, 95% CI
ATTRACTION 3	30.2%	1.08 [0.87, 1.34]
KEYNOTE 061	34.0%	1.49 [1.25, 1.78]
KEYNOTE 181	35.8%	1.11 [0.94, 1.29]
**Total (95% CI)**	**100.0%**	**1.22 [0.99, 1.49]**

Heterogeneity: Tau² = 0.02; Chi² = 7.69, df = 2 (P = 0.02); I² = 74%
Test for overall effect: Z = 1.87 (P = 0.06)

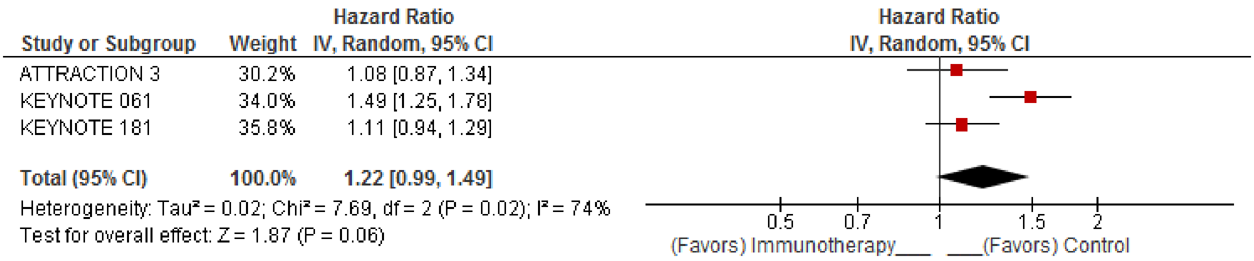

Supplementary Figure 10. Pooled Odds Ratio for Objective Response Rate of 2nd line studies evaluating immune checkpoint inhibitors in upper gastrointestinal malignancies. The error bars represent the 95% confidence intervals. A 2-sided p value < .05 was considered significant.

A) 2nd line, all studies were included; B) 2nd line, patients with any PD-L1 CPS ≥ 1% were included; C) 2nd line, patients with PD-L1 CPS ≥ 10% were included; D) 2nd line, patients with adenocarcinomas were included; E) 2nd line, patients with adenocarcinomas and PD-L1 CPS ≥ 10% were included; F) 2nd line, only patients with squamous cell carcinomas were included

The Mantel-Haenszel (MH) statistical method was applied for calculation of pooled ORs. In case of statistically significant heterogeneity (Q test P<0.1) the Random Effects (RE) model was reported; otherwise, the Fixed Effects (FE) model was adopted to estimate the pooled ratios.

Abbreviations: PD-L1: Programmed death ligand 1; CPS: Combined positive score; MH: Mantel-Haenszel; OR: Odds Ratio; CI: confidence intervals; FE: Fixed Effects; RE: Random Effects.

A.

	Immunotherapy&Chemo		Chemo			Odds Ratio
**Study or Subgroup**	**Events**	**Total**	**Events**	**Total**	**Weight**	**M-H, Random, 95% CI**
ATTRACTION 3	33	171	34	158	21.6%	0.87 [0.51, 1.49]
ESCORT	46	228	14	220	20.4%	3.72 [1.98, 6.99]
KEYNOTE 061	36	296	45	296	22.3%	0.77 [0.48, 1.24]
KEYNOTE 063	6	47	9	47	14.3%	0.62 [0.20, 1.90]
KEYNOTE 181	41	314	21	314	21.4%	2.10 [1.21, 3.64]
**Total (95% CI)**		**1056**		**1035**	**100.0%**	**1.31 [0.69, 2.49]**
Total events	162		123			

Heterogeneity: Tau² = 0.42; Chi² = 22.11, df = 4 (P = 0.0002); I² = 82%
Test for overall effect: Z = 0.82 (P = 0.41)

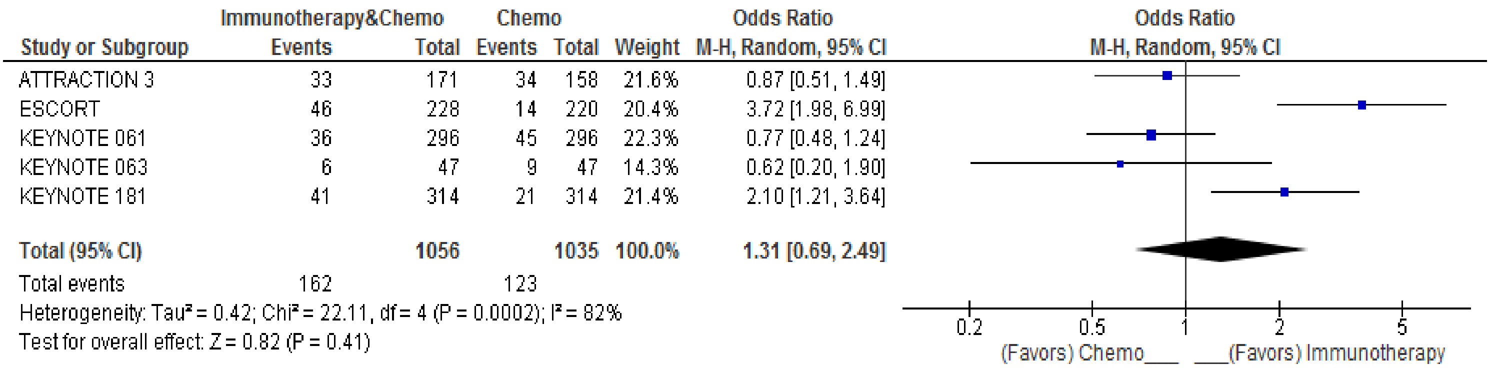

B.

	Immunotherapy&Chemo		Chemo			Odds Ratio
**Study or Subgroup**	**Events**	**Total**	**Events**	**Total**	**Weight**	**M-H, Fixed, 95% CI**
KEYNOTE 061	32	196	27	199	74.1%	1.24 [0.71, 2.17]
KEYNOTE 063	6	47	9	47	25.9%	0.62 [0.20, 1.90]
**Total (95% CI)**		**243**		**246**	**100.0%**	**1.08 [0.66, 1.77]**
Total events	38		36			

Heterogeneity: Chi² = 1.20, df = 1 (P = 0.27); I² = 16%
Test for overall effect: Z = 0.31 (P = 0.76)

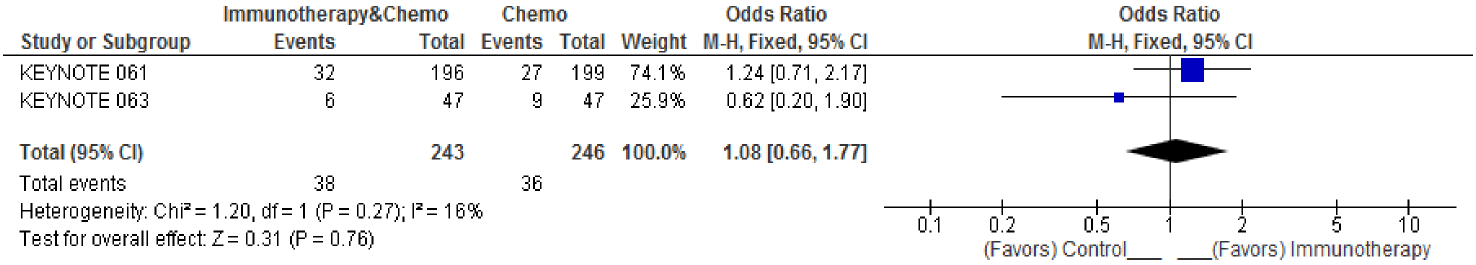

C.

	Immunotherapy&Chemo		Chemo			Odds Ratio
**Study or Subgroup**	**Events**	**Total**	**Events**	**Total**	**Weight**	**M-H, Fixed, 95% CI**
KEYNOTE 061	13	53	5	55	41.1%	3.25 [1.07, 9.88]
KEYNOTE 181	23	107	7	115	58.9%	4.22 [1.73, 10.32]
**Total (95% CI)**		**160**		**170**	**100.0%**	**3.82 [1.91, 7.66]**
Total events	36		12			

Heterogeneity: Chi² = 0.13, df = 1 (P = 0.72); I² = 0%
Test for overall effect: Z = 3.78 (P = 0.0002)

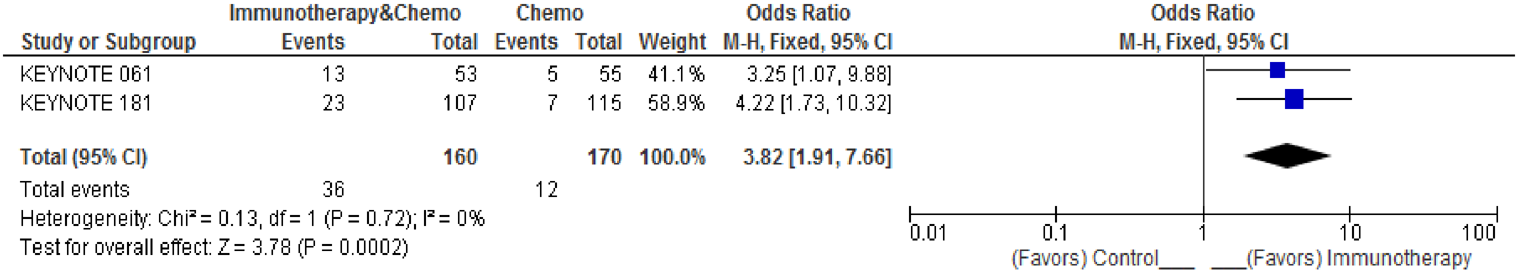

D.

Study or Subgroup	IO&Chemo Events	IO&Chemo Total	Chemo Events	Chemo Total	Weight	Odds Ratio M-H, Fixed, 95% CI
KEYNOTE 061	36	296	45	296	83.4%	0.77 [0.48, 1.24]
KEYNOTE 063	6	47	9	47	16.6%	0.62 [0.20, 1.90]
**Total (95% CI)**		**343**		**343**	**100.0%**	**0.75 [0.48, 1.15]**
Total events	42		54			

Heterogeneity: Chi² = 0.13, df = 1 (P = 0.72); I² = 0%
Test for overall effect: Z = 1.32 (P = 0.19)

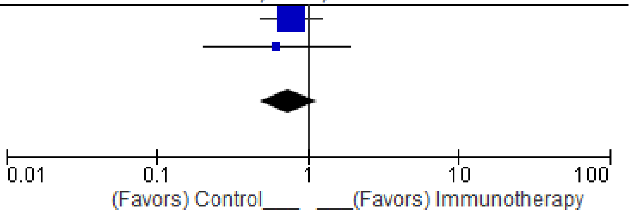

E.

Study or Subgroup	IO&Chemo Events	IO&Chemo Total	Chemo Events	Chemo Total	Weight	Odds Ratio M-H, Fixed, 95% CI
KEYNOTE 061	13	53	5	55	85.0%	3.25 [1.07, 9.88]
KEYNOTE 181	4	22	1	33	15.0%	7.11 [0.74, 68.56]
**Total (95% CI)**		**75**		**88**	**100.0%**	**3.83 [1.42, 10.32]**
Total events	17		6			

Heterogeneity: Chi² = 0.37, df = 1 (P = 0.54); I² = 0%
Test for overall effect: Z = 2.65 (P = 0.008)

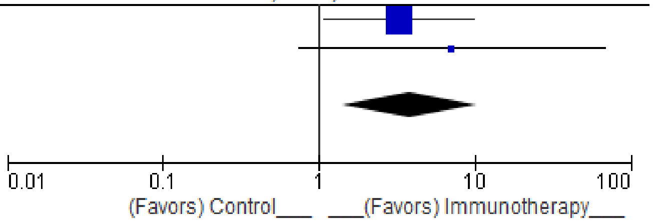

F.

Study or Subgroup	IO&Chemo Events	IO&Chemo Total	Chemo Events	Chemo Total	Weight	Odds Ratio M-H, Random, 95% CI
ATTRACTION 3	33	171	34	158	34.5%	0.87 [0.51, 1.49]
ESCORT	46	228	14	220	32.9%	3.72 [1.98, 6.99]
KEYNOTE 181	33	198	15	203	32.6%	2.51 [1.31, 4.78]
**Total (95% CI)**		**597**		**581**	**100.0%**	**1.98 [0.81, 4.84]**
Total events	112		63			

Heterogeneity: Tau² = 0.53; Chi² = 13.12, df = 2 (P = 0.001); I² = 85%
Test for overall effect: Z = 1.51 (P = 0.13)

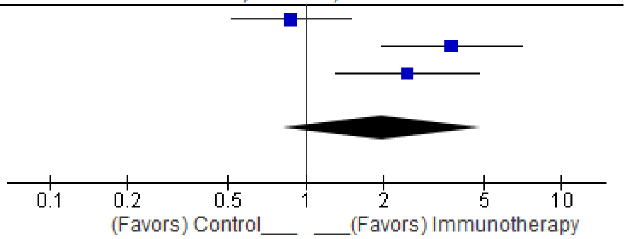

Supplementary Figure 11. Pooled Hazard Ratio for overall survival of 1st line studies evaluating immune checkpoint inhibitors in upper gastrointestinal malignancies in MSI High patients. The error bars represent the 95% confidence intervals. A 2-sided p value < .05 was considered significant.

A) 1st line, both monotherapy and combined immunotherapy with chemotherapy arms included; B) 1st line, combined chemotherapy-immunotherapy arm included.

The Inverse-Variance (IV) statistical method was applied for calculation of pooled Hazard Ratios (HRs No heterogeneity was detected, so the Fixed Effects (FE) model was adopted to estimate the pooled ratio.

Abbreviations: MSI: Microsatellite instability; IV: Inverse-Variance; HR: Hazard Ratio; CI: confidence intervals; FE: Fixed Effects; IO: immunotherapy.

A.

Study or Subgroup	Weight	Hazard Ratio IV, Fixed, 95% CI
CHECKMATE 649	34.7%	0.33 [0.13, 0.84]
KEYNOTE 062 (combinedIO)	32.0%	0.37 [0.14, 0.99]
KEYNOTE 062 (monotherapy IO)	33.3%	0.29 [0.11, 0.76]
**Total (95% CI)**	**100.0%**	**0.33 [0.19, 0.57]**

Heterogeneity: Chi² = 0.13, df = 2 (P = 0.94); I² = 0%
Test for overall effect: Z = 3.94 (P < 0.0001)

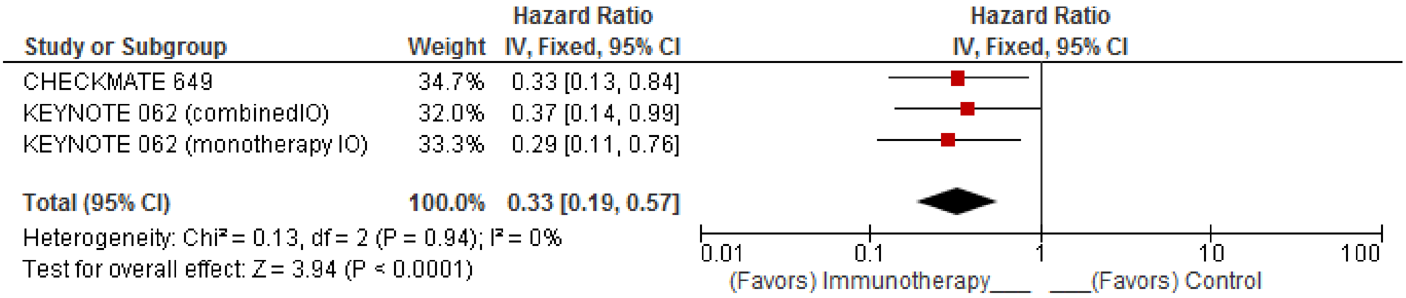

B.

Study or Subgroup	Weight	Hazard Ratio IV, Fixed, 95% CI
CHECKMATE 649	52.0%	0.33 [0.13, 0.84]
KEYNOTE 062 (combinedIO)	48.0%	0.37 [0.14, 0.99]
**Total (95% CI)**	**100.0%**	**0.35 [0.18, 0.69]**

Heterogeneity: Chi² = 0.03, df = 1 (P = 0.86); I² = 0%
Test for overall effect: Z = 3.04 (P = 0.002)

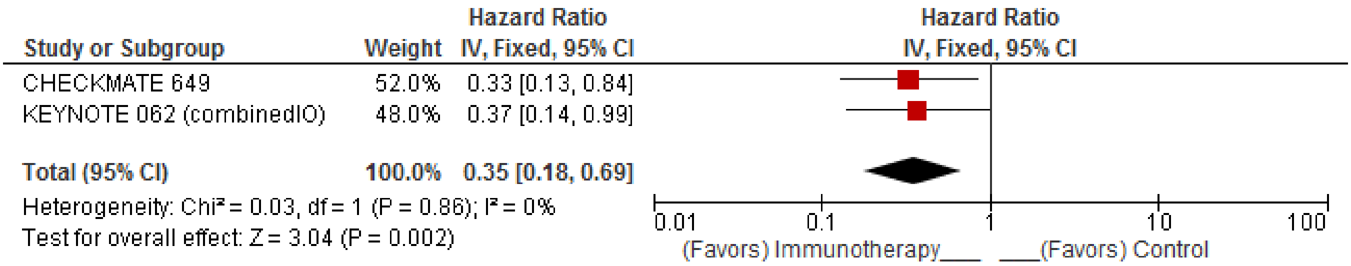

Supplement: pkab088_Supplementary_Data [file pkab088_supplementary_data.pdf]
